# Early Prediction of Cardiac Arrest in the Intensive Care Unit Using Explainable Machine Learning: Retrospective Study

**DOI:** 10.2196/62890

**Published:** 2024-09-17

**Authors:** Yun Kwan Kim, Won-Doo Seo, Sun Jung Lee, Ja Hyung Koo, Gyung Chul Kim, Hee Seok Song, Minji Lee

**Affiliations:** 1 Technology Development Seers Technology Co. Ltd. Pyeongtaek-si, Gyeonggi-do Republic of Korea; 2 Department of Brain and Cognitive Engineering Korea University Seoul Republic of Korea; 3 Department of Biomedical Software Engineering The Catholic University of Korea Bucheon-si, Gyeonggi-do Republic of Korea

**Keywords:** early cardiac arrest warning system, electric medical record, explainable clinical decision support system, pseudo-real-time evaluation, ensemble learning, cost-sensitive learning

## Abstract

**Background:**

Cardiac arrest (CA) is one of the leading causes of death among patients in the intensive care unit (ICU). Although many CA prediction models with high sensitivity have been developed to anticipate CA, their practical application has been challenging due to a lack of generalization and validation. Additionally, the heterogeneity among patients in different ICU subtypes has not been adequately addressed.

**Objective:**

This study aims to propose a clinically interpretable ensemble approach for the timely and accurate prediction of CA within 24 hours, regardless of patient heterogeneity, including variations across different populations and ICU subtypes. Additionally, we conducted patient-independent evaluations to emphasize the model’s generalization performance and analyzed interpretable results that can be readily adopted by clinicians in real-time.

**Methods:**

Patients were retrospectively analyzed using data from the Medical Information Mart for Intensive Care-IV (MIMIC-IV) and the eICU-Collaborative Research Database (eICU-CRD). To address the problem of underperformance, we constructed our framework using feature sets based on vital signs, multiresolution statistical analysis, and the Gini index, with a 12-hour window to capture the unique characteristics of CA. We extracted 3 types of features from each database to compare the performance of CA prediction between high-risk patient groups from MIMIC-IV and patients without CA from eICU-CRD. After feature extraction, we developed a tabular network (TabNet) model using feature screening with cost-sensitive learning. To assess real-time CA prediction performance, we used 10-fold leave-one-patient-out cross-validation and a cross–data set method. We evaluated MIMIC-IV and eICU-CRD across different cohort populations and subtypes of ICU within each database. Finally, external validation using the eICU-CRD and MIMIC-IV databases was conducted to assess the model’s generalization ability. The decision mask of the proposed method was used to capture the interpretability of the model.

**Results:**

The proposed method outperformed conventional approaches across different cohort populations in both MIMIC-IV and eICU-CRD. Additionally, it achieved higher accuracy than baseline models for various ICU subtypes within both databases. The interpretable prediction results can enhance clinicians’ understanding of CA prediction by serving as a statistical comparison between non-CA and CA groups. Next, we tested the eICU-CRD and MIMIC-IV data sets using models trained on MIMIC-IV and eICU-CRD, respectively, to evaluate generalization ability. The results demonstrated superior performance compared with baseline models.

**Conclusions:**

Our novel framework for learning unique features provides stable predictive power across different ICU environments. Most of the interpretable global information reveals statistical differences between CA and non-CA groups, demonstrating its utility as an indicator for clinical decisions. Consequently, the proposed CA prediction system is a clinically validated algorithm that enables clinicians to intervene early based on CA prediction information and can be applied to clinical trials in digital health.

## Introduction

Critical illness is defined as the presence or potential development of organ dysfunction. Cardiac arrest (CA), a critical condition that impacts patient safety, refers to the sudden cessation of cardiac function due to specific abnormal events, such as ventricular arrhythmia, asystole, and pulseless electrical activity [1]. At least one abnormal sign, such as respiratory distress or hemodynamic instability, occurs in 59.4% of patients within 1-4 hours before the onset of CA [2]. Early identification of the causes of CA improves patient survival by approximately 29% within the first hour of the episode and 19% at discharge [3]. Therefore, early prediction of CA is crucial to allow for more time for clinical intervention, thereby reducing mortality.

Clinical decision support systems (CDSSs) are clinical computer systems that apply algorithms to patient information, use machine learning to evaluate clinical data, and provide clinical decision support [4,5]. These systems, developed using electronic medical records, utilize various paradigms—such as predicting early cardiac events, heart failure (HF), and critical illness—to enable rapid response through real-time patient monitoring [6-9]. To enhance the quality and speed of medical services, CA prediction and warning systems have been developed for use in intensive care units (ICUs) within the field of CDSSs [6]. These computer-based CA prediction algorithms offer new opportunities for clinicians to improve the accuracy of predicting CA events [10].

Traditional score–based methods, including the Simplified Acute Physiology Score (SAPS)-II, Sequential Organ Failure Assessment (SOFA), and Modified Early Warning Score (MEWS), are tools used by in-hospital care teams to identify early indicators of CA and initiate early intervention and therapy [11-15]. However, these score-based systems suffer from low sensitivity or a high false alarm rate [16]. To address these issues, machine learning methods have been used in CA prediction [17,18], leading to significant improvements in performance.

Churpek et al [19] proposed the use of a random forest (RF) classifier, based on clinical information extracted from a multicenter data set, achieving an area under the receiver operating characteristic curve (AUROC) of 0.83. Similarly, Hong et al [20] implemented an RF model using a clinical data set from a retrospective study, attaining an AUROC of 0.97 and an area under the precision-recall curve (AUPRC) of 0.86. Although the authors achieved accurate CA prediction results, their methodology relied heavily on features not commonly used during hospitalization and did not offer real-time predictions. To address this, Layeghian Javan et al [21] proposed a stacking method that combines RF, balanced bagging, and logistic regression to predict CA 1 hour in advance. Layeghian Javan et al [21] achieved an AUROC of 0.82 using the Medical Information Mart for Intensive Care (MIMIC)-III data set. Kwon et al [17] proposed a deep learning–based early warning system that utilizes a recurrent neural network (RNN) to assess risk scores from input vectors measured over an 8-hour period. Their system, based on vital signs extracted from a retrospective multicenter cohort data set, resulted in AUROC and AUPRC values of 0.85 and 0.04, respectively. Additionally, Kim et al [18] developed an ensemble-based CA prediction system using the light gradient boosting method (LGBM) to predict CA 1 hour in advance, obtaining AUROC and AUPRC values of 0.86 and 0.58, respectively, using the MIMIC-IV data set.

As mentioned earlier, artificial intelligence (AI) has been applied to CA prediction solutions and has demonstrated high predictive power in several studies [22]. However, hospitals often group patients with similar conditions and illness severity within the same unit for more efficient treatment. Specifically, ICUs are divided into subtypes, such as general ICUs and cardiac ICUs, to optimize care. In this context, previous studies on CA prediction focused on the entire ICU without accounting for the heterogeneity within subtypes. As a result, the performance of CA prediction models may vary depending on the distinct characteristics of each group [23].

Although numerous studies have applied AI to CA prediction [17,18], challenges persist in their practical application. First, CA prediction studies must confirm clinical validity through multicenter studies [2]. However, clinical maturity for CA prediction has not been established when monitored in real-time, as validation was typically performed using representative events extracted from the validation site. Second, patients grouped into different ICU subtypes exhibit varying characteristics and likelihood of developing CA. However, the performance of CA prediction models across these subtypes has not been validated. Third, while interpreting the results of prediction models is crucial for clinicians to make informed decisions [13], an interpretable model capable of providing this information in real-time monitoring—especially among deep learning–based models—has yet to be developed.

This study proposes a framework for early and accurate prediction of CA across diverse clinical settings, accounting for heterogeneity. We aim to validate the clinical maturity, safety, and effectiveness of the proposed framework by comparing it with existing trigger systems and machine learning methods using a pseudo real-time CA evaluation system. We propose a novel framework that learns patient-independent and subtype-specific characteristics in the ICU to improve CA prediction and reduce the false alarm rate. As a deep learning–based model optimized for tabular data, such as tabular network (TabNet), it can address the overfitting and performance limitations of existing tree-based models [24]. In addition, a cost-sensitive learning approach was applied to address the class imbalance in CA events. We then used the MIMIC-IV and eICU-Collaborative Research Database (eICU-CRD) to evaluate clinical maturity across various patient populations and ICU subtypes [25,26]. To illustrate changes in feature importance over time for clinical decisions, we utilized the MIMIC-IV data set [25]. Therefore, the proposed CA prediction framework can offer clinicians a reliable warning of CA occurrence within 24 hours. It also provides interpretable information about CA alarms and insights for rapid response.

## Methods

### Data Source

We used 2 databases: MIMIC-IV and eICU-CRD. The MIMIC-IV database, which includes information on vital signs, laboratory tests, and procedural events for ICU patients, was utilized to develop and validate a CA prediction model using multivariate vital sign time-series data from patients with HF. Specifically, MIMIC-IV is a well-known single-center database containing information on 46,520 patients admitted to the Beth Israel Deaconess Medical Center (BIDMC) between 2008 and 2019. The database includes demographic data, International Classification of Diseases (ICD) codes, clinical modification codes, hourly vital signs, inputs and outputs, laboratory test and microbiological culture results, imaging data, treatment methods, medication administration, and survival statistics. In addition, MIMIC-IV includes data from the clinical information system iMDsoft MetaVision. Compared with MIMIC-III, which extracts data from heterogeneous sources, MIMIC-IV provides more comprehensive patient data and detailed information on procedural events, serving as a primary source of clinical information in ICUs [25]. Consequently, MIMIC-IV data are more homogeneous compared with MIMIC-III data [25].

The eICU-CRD contains data from over 200,000 ICU admissions monitored across the United States through the eICU-CRD program developed by Philips Healthcare. This collaborative database includes information on patients admitted to the ICU in 2014 and 2015 [26].

### Ethical Considerations

The MIMIC-IV database and eICU-CRD are deidentified, transformed, and made available to researchers who have completed human research training and signed a data use agreement. The Institutional Review Board at the BIDMC granted a waiver of informed consent and approved the sharing of the MIMIC-IV database. Similarly, the eICU-CRD data were exempt from Institutional Review Board approval and were also granted a waiver of informed consent [25,26].

To enhance the system for doctors and patients, AI has addressed concerns related to bias and fairness in health care. Our strategies to mitigate these issues are as follows:

Bias in AI generally arises from 2 primary sources: the data used for algorithmic training (data bias) and the intrinsic design or learning mechanisms of the algorithm itself (algorithmic bias). In health care settings, the involvement of human interaction and decision-making can introduce additional bias due to the inherently complex nature of the process [27]. To mitigate the impact of data bias, we used a patient-centered data set rather than relying solely on representative event data. Additionally, we conducted a subgroup analysis to identify potential biases in various environments. Key factors contributing to algorithmic bias are label bias and cohort bias [28,29]. Label bias has been addressed through updates to the MIMIC-IV and eICU-CRD databases [25,26]. To counter cohort bias, which may arise when different group levels are not adequately considered, we evaluated both patients with heart disease and the broader population using different databases [30].

Fairness in health care is multidimensional, involving the equitable distribution of resources, opportunities, and outcomes among diverse patient populations. Health care systems must ensure access to quality care for all individuals without discrimination [31]. To uphold this fairness, we selected MIMIC-IV and eICU-CRD—2 representative databases in critical care—and proceeded with AI development only after minimizing bias in each database. Additionally, we assessed explainability and the Brier score to address potential errors, harmful outcomes, and biases in AI-generated predictions.

The use of AI-driven predictions in critical care decisions can introduce various biases. Bias related to clinician interaction may be common in CDSS systems, with risks including overconfidence in the AI system or desensitization to real-world events due to excessive alerts [32,33]. To mitigate these biases, we evaluated the false alarm rate, event recall, and sensitivity. Additionally, future deployments will require clinician training on inherent biases and regular monitoring of the algorithm.

### Problem Definition

The task of the study is to predict CA events within 24 hours. The input data include the patient’s vital signs and the MEWS over a 12-hour time window. We then generate continuous labels every hour regarding the risk of CA within the next 24 hours and calculate the alarm rate based on the correctness of the alarms. The primary outcome was the AUROC, used to quantitatively assess the prediction results for CA events within 24 hours. The alarm rate, including false alarm rate and event recall, was calculated as a secondary outcome to evaluate alarm fatigue. Sensitivity was also assessed as a secondary outcome to identify any reductions in false alarms or missed CA events. Additionally, we provided clinically interpretable decision support information.

### Prediction Model Framework

#### Overview

**Figure 1:**
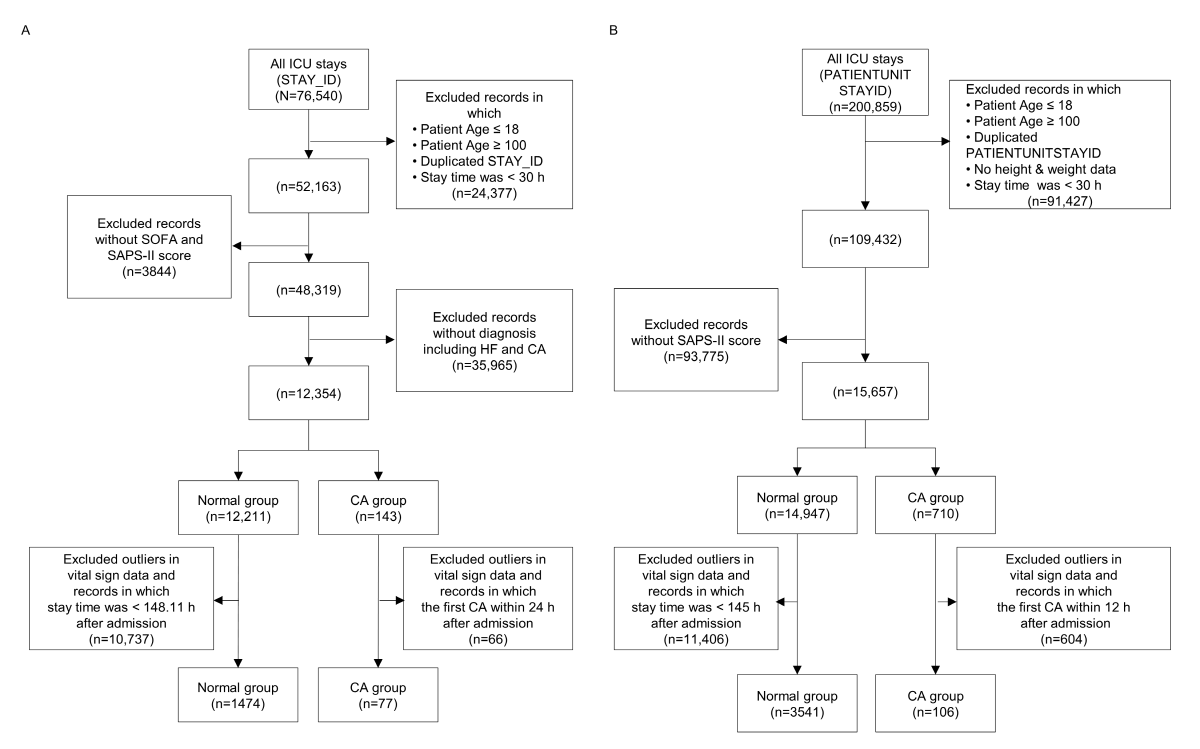
Patient inclusion and exclusion flow diagram for the MIMIC-IV and eICU-CRD. (A) MIMIC-IV, (B) eICU-CRD. CA: cardiac arrest; eICU-CRD: eICU-Collaborative Research Database; HF: heart failure; ICU: intensive care unit; MIMIC: Medical Information Mart for Intensive Care; SAPS: Simplified Acute Physiology Score; SOFA: Sequential Organ Failure Assessment.

We propose a framework for predicting CA events within 24 hours in advance. As illustrated in Figure 1, the framework consists of 6 components: data preparation, data preprocessing and extraction, feature generation, feature aggregation and CA event labeling, model development, and evaluation. Details about the open-source tools and development code used are provided in Multimedia Appendix 1 [34].

After applying the inclusion and exclusion criteria, we extracted vital signs and calculated the MEWS based on these vital signs. In step 2, we processed and normalized the features after resampling the vital signs and MEWS to a 1-hour resolution. In step 3, we generated multiresolution statistical features and Gini index–based features. The multiresolution statistical features were created using a sliding window approach to segment each vital sign into 4-, 6-, and 12-hour intervals. Next, we generated continuous labels every hour indicating the risk of CA within the next 24 hours. In step 4, we aggregated multiresolution statistical features, Gini index–based features, and labels. In step 5, we developed a TabNet classifier, known for its effectiveness in various classification tasks with tabular data [24], and incorporated different cost weights for each class. Finally, in step 6, we evaluated the performance of the proposed model using sensitivity, false alarm rate, event recall, and AUROC.

#### Step 1: Data Preparation

**Figure 2:**
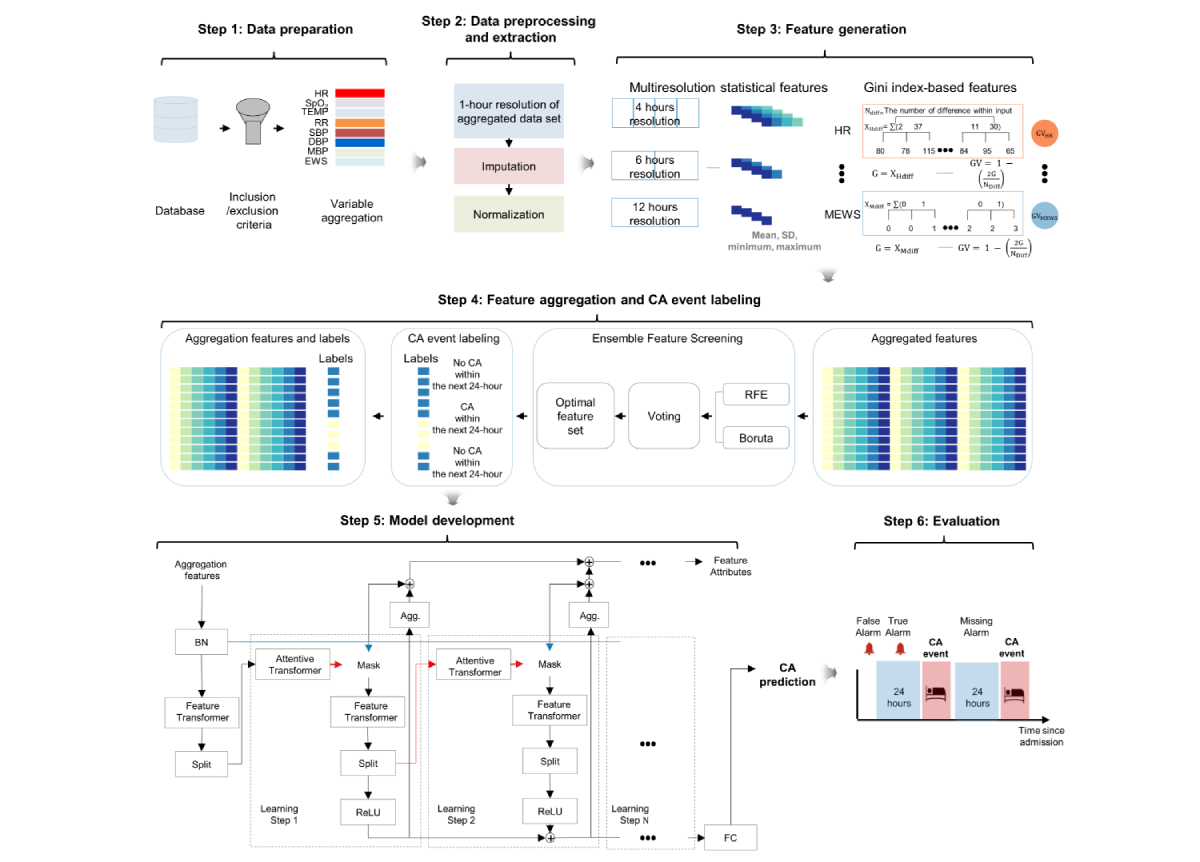
Overview of the proposed framework. This is composed of 6 steps including data preparation; data preprocessing and extraction; feature generation; feature aggregation and CA event labeling; model development; and evaluation. Three components make up TabNet, including feature transformer, attentive transformer, and feature masking. A split block separates the processed representation for the overall output and is used by the attentive transformer of the next phase. The feature selection mask provides comprehensible details about the functioning of the model for each step, and the masks can be combined to produce global feature important attribution. BN: batch normalization; CA: cardiac arrest; DBP: diastolic blood pressure; EWS: early warning score; HR: heart rate; MBP: mean blood pressure; MEWS: Modified Early Warning Score; ReLU: rectified linear unit; RFE: recursive feature elimination; RR: respiratory rate; SBP: systolic blood pressure; SpO_2_: oxyhemoglobin saturation; TabNet: tabular network; TEMP: temperature.

Data were obtained from the MIMIC-IV and eICU-CRD databases to construct cohorts meeting the inclusion and exclusion criteria [25]. The target populations of the 2 databases differed: MIMIC-IV includes only patients with cardiac issues in the ICU, while eICU-CRD encompasses all ICU patients. The number of CA events per patient also varied between the databases. MIMIC-IV typically has 1 CA event per patient, whereas eICU-CRD often records multiple CA events per patient. As CA events can occur multiple times per patient in a clinical setting, we validated multiple events in the eICU-CRD. Finally, we performed an analysis that accounted for differences across databases to compare the performance of CA prediction between high-risk patient groups and those without CA, as well as across different clinical settings, using the proposed framework, as shown in Figure 2.

For the inclusion and exclusion processing of MIMIC-IV, we applied the criteria to select the study cohort. Patients aged over 18 and under 100 years were included. Records of patients without SOFA and SAPS-II scores were excluded, as these scores were used to compare the prediction performance of the prognostic scales with the proposed framework. HF is a major risk factor for sudden CA and a significant contributor to CA-related mortality. CA is more prevalent in patients with a history of HF or previous CA. Therefore, we included ICU stays of patients with these cardiovascular conditions in the cohort study. For the CA group, we included ICU data if the vital sign data were not outliers and if any events occurred within 1 hour before the CA within 24 hours of patient admission.

For the inclusion and exclusion processing of the eICU-CRD, we acknowledged the differences in target population characteristics as mentioned above. Therefore, the inclusion and exclusion criteria were the same as those for MIMIC-IV, except for the criteria related to patients with high-risk CA.

MIMIC-IV includes only patients with cardiac issues in the ICU, while eICU-CRD encompasses all ICU patients. Consequently, the MIMIC-IV data set included 77 patients in the CA group and 1474 patients in the normal group, whereas the eICU-CRD data set included 106 patients in the CA group and 3641 patients in the normal group.

#### Step 2: Data Preprocessing and Extraction

We collected data on vital sign parameters, including heart rate (HR), systolic blood pressure (SBP), diastolic blood pressure (DBP), temperature, respiratory rate (RR), and oxyhemoglobin saturation (SpO_2_) from the experimental database. These vital sign parameters may be recorded with irregular time-series data due to equipment malfunctions and varying patient responses [35]. Prediction models are not designed to classify data with irregular time-series samples between groups. To address this issue, the models require data collected at regular time intervals. We used a bucketing technique to manage the irregularities in the time series [21]. We divided the 12-hour time windows into 12 sequential 1-hour buckets, and the measured values within each bucket were averaged. Consequently, each time series consisted of 12 values at regular 1-hour intervals. If there were no values in a bucket, it was marked as null. To address missing values, we used the last observation carried forward (LOCF) and last observation carried backward (LOCB) imputation techniques [36]. In the LOCF method, missing values are filled by carrying forward the most recent nonmissing values. Similarly, in the LOCB method, missing values are filled by carrying backward the subsequent nonmissing values. Although we primarily used the LOCB method to impute missing values, the LOCF method was applied when subsequent values were missing, filling in missing values with the most recent nonmissing values.

Additionally, we extracted the early warning scores (EWS) for vital signs. We used the MEWS [15], a composite score commonly used by medical staff to assess illness severity. EWS observations were assigned scores ranging from 0 to 3. The EWS was calculated every hour. To remove outliers, we determined the acceptable range for each variable based on the input from medical experts. Values falling outside this range were eliminated. We normalized each feature using the minimum and maximum values within the abnormal range for each vital sign, as each feature column had a different scale. We converted the database into an hourly time series with 12-hour intervals. Subsequently, we combined the CA and non-CA groups to perform the imputation task.

#### Step 3: Feature Generation and Aggregation

##### Feature Extraction and Processing for Cardiac Arrest Prediction

After applying the inclusion and exclusion criteria, we extracted vital signs—such as HR, SBP, DBP, temperature, RR, and SpO_2_—and calculated the MEWS based on these vital signs. The features were then processed and normalized after resampling both the vital signs and MEWS to a 1-hour resolution [37]. The database was organized into an hourly time series with 12-hour intervals. Finally, we combined the CA and non-CA groups to perform the imputation task.

We generated 3 types of features within a 12-hour time window: vital sign–based features, multiresolution statistical features, and Gini index–based features. These features were designed to capture meaningful changes for predicting the occurrence of CA by identifying temporal patterns in vital signs, statistical variations across different resolutions, and the degree of information imbalance. The method for generating these features is outlined in the following sections.

##### Vital Sign–Based Features

To extract the pattern of vital signs, we used normalized vital signs and MEWS within a 12-hour time window.

##### Multiresolution Statistical Features

To capture statistical changes across different sections, we created time windows of increasing sizes and extracted summary statistics from these multiresolution sliding windows. For the multiresolution sliding window–based statistical features, the input data were segmented into resolutions of 0-4 hours, 0-6 hours, and 0-12 hours. Each time-series segment of the vital sign data was then aggregated to calculate the mean, median, minimum, maximum, and SD for each feature.

Specifically, the temporal patterns of each biological signal over a 12-hour period, with representative values for each 1-hour segment, show distinctive characteristics between the group that experienced CA and the group that did not. However, compressing the 5 statistical features mentioned above for the entire input window into a single statistical summary may not fully capture the differences in patterns between the groups. Consequently, 5 statistical values were derived by shifting a 4-hour window across the 12-hour input window. This approach allowed us to obtain statistical values for each section using both 6- and 12-hour windows. This feature extraction method, previously used in our research, was shown to enhance CA prediction accuracy by providing a condensed statistical summary of various sections [18].

##### Gini Index–Based Features

Inspired by the Gini index, which measures statistical variance to indicate income inequality in economics, we propose a method to calculate the imbalance of patterns within each vital sign over the input time steps. This method calculates inequality for each vital sign and uses it as a feature to distinguish between situations where CA occurred and did not occur. Previous research suggests that significant changes in temporal patterns often precede CA, making this imbalance a valuable characteristic variable. The index-based feature formulation is expressed as follows:



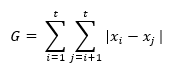



GV = 1 – (2G/N_Diff_)

where G is the index; GV indicates index-based features of each vital sign; x_i_ and x_j_ are the values of vital signs within the input range in each vital sign; and N_Diff_ is the number of intervals in the input vital sign. We calculated the Gini index to assess the impurity of each vital sign variation and then performed the normalization step, denoted as GV.

For instance, if the Gini index–based feature value is relatively low, it indicates that the values within the input window are stable. Conversely, if there is a rapid change in HR before CA, the Gini index–based feature value will increase. This pattern change is captured by the Gini index, which measures statistical dispersion.

#### Step 4: Feature Aggregation and CA Event Labeling

We aggregated multiresolution statistical features, Gini index–based features, and labels to enhance temporal features and achieve better inter-ICU generalizations from the model, utilizing vital signs and specific clinical latent scores.

To select and screen the most relevant and nonredundant features, we used 2 feature selection methods: recursive feature elimination and the Boruta method. Recursive feature elimination identifies the most relevant features for predicting the target by recursively eliminating a small number of features in each iteration [38]. This process helps eliminate collinearity within the proposed framework. The Boruta method assesses the relevance of each feature using statistical testing and shadow features [39].

To overcome the limitations of conventional feature selection methods, we used an ensemble feature screening approach using a majority voting mechanism. This method was applied to a total of 653 features, resulting in the selection of 86 features from the MIMIC-IV database and 94 features from the eICU-CRD database. In this approach, each feature screening method casts 1 vote for a selected feature, and a feature will receive 2 votes if both feature screening methods choose it.

We then aggregated the variables into binary indicators to denote the presence or absence of CA in each class.

#### Step 5: Model Development

We used a TabNet classifier with features from a 12-hour time window to predict CA events within a 24-hour period. We generated continuous labels every hour indicating the risk of CA in the next 24 hours and calculated the alarming rate based on whether the alarm was correct or incorrect. Additionally, we applied cost-sensitive learning as an algorithm-level approach to address the extreme imbalances in the MIMIC-IV and eICU-CRD data sets [40]. Cost-sensitive learning was applied to penalize errors in the minority class (the CA group). The TabNet classifier, using cost-sensitive learning, helps reduce bias or variance and improve the stability of machine learning algorithms [41,42]. The minority classes from the MIMIC-IV and eICU-CRD data sets were penalized with a weight of 100. Finally, we set the weight for the CA class to 100 and the learning rate to 0.01.

#### Step 6: Evaluation

##### Leave-One-Patient-Out K-Fold Internal Validation

We used leave-one-patient-out (LOPO) K-fold validation to evaluate the individual patient performance of CA prediction and to provide a realistic estimate of model performance on new patients [43]. This method is a variant of K-fold cross-validation, where the folds consist of individual patients. Specifically, we used a 10-fold LOPO validation [44].

Additionally, we established a pseudo-real-time CA evaluation system to assess and compare the real-time CA prediction performance for hospitalized patients, simulating an ICU environment. This system used the LOPO K-fold validation approach to predict CA events within a 24-hour period.

##### Cross-Data Set External Validation

In this experimental setting, both the proposed method and comparison methods were trained on 1 database and tested on another to evaluate their generalization ability [45]. We alternately used MIMIC-IV and eICU-CRD as the source and target databases to assess generalization performance. To ensure consistency in feature properties across databases, we excluded SAPS-II, which includes laboratory tests. The experiment consisted of 2 steps. First, we trained the models, including the proposed method and comparison models, using MIMIC-IV as the source database. Next, we tested the CA prediction performance on eICU-CRD using the trained models and compared their performance.

To assess the generalization ability and minimize the impact of specific group characteristics in the learning data, we conducted another cross-data set external validation. We trained the models using eICU-CRD as the source database and evaluated CA prediction performance on MIMIC-IV as the target database.

##### Subgroup Analysis

We evaluated the CA prediction performance for each ICU subtype to determine if there were differences in performance between the proposed method and the comparative models for each ICU cluster. The common ICU subtypes across MIMIC-IV and eICU-CRD were general, cardiac, neuro, and trauma. Neuro and trauma ICUs were excluded from this analysis due to the low number or absence of admissions of patients with CA. We used 10-fold LOPO cross-validation for subgroup evaluation. In this process, the entire data set assigned to each fold was used for training, and during testing, performance was evaluated by categorizing patients according to their ICU subtype.

##### Baseline Models

To evaluate the performance of the proposed method, we used the following baseline models: National Early Warning Score (NEWS), SOFA, SAPS-II, logistic regression, k-nearest neighbors, multilayer perceptron, LGBM, RNN, and reverse time attention. Details of these baseline models can be found in Multimedia Appendix 2.

##### Evaluation Metrics

We assessed the performance of the proposed and baseline methods using AUROC, event recall, false alarm rate, and sensitivity. Our goal was to evaluate the proposed method in a clinically relevant context by focusing on the percentage of CA events detected and the rate of false alarms. We specifically measured event recall [46], which quantifies whether the CA prediction system correctly triggered an alarm in the period preceding a CA event.

ER = *N*_Captured_/*N*_Total_

where ER is event recall; *N*_Captured_ implies the number of captured events; and *N*_Total_ indicates the number of total CA events.

Next, the false alarm rate was defined as the fraction of alarms that failed to detect an actual event, to investigate whether operational costs were being wasted. This concept is analogous to exon prediction in gene discovery. The false alarm rate is evaluated as follows:

FAR = 1 – (*N*_True_/*N*_Alarm_)

where FAR is the false alarm rate; *N*_True_ is the number of true alarms; and *N*_Alarm_ is the number of total alarms in the CA prediction system.

### Explainable Predictions

We extracted both local and global interpretability information by examining the decision masks of TabNet. After determining the impact of each feature using the proposed model, we summarized and visualized the top 25 features with the highest mean values. Additionally, we visualized the impact of features over time using a heatmap and tracked changes in the features with the highest values. To compare the differences in impact between non-CA and CA groups, we conducted a statistical test. Specifically, an independent *t* test with false discovery rate (FDR) correction was used to assess the differences in interpretability information between the 2 groups.

### Statistical Analysis

Differences in patient characteristics, such as age, ICU length of stay, and vital signs, between the non-CA and CA groups were evaluated using independent *t* tests. To compare performance metrics between the baseline and proposed models, we used the Kruskal-Wallis test, followed by the honestly significant difference (HSD) test for post hoc analysis. The differences in interpretable information between the non-CA and CA groups were evaluated using an independent *t* test with FDR correction. A significance level of 5% (*P*<.05) was used for all analyses.

## Results

### Patient Characteristics

The patient characteristics are presented as means and SDs in Table 1.

In the 12-hour time window for MIMIC-IV, age did not differ significantly between the CA and non-CA groups. However, the ICU length of stay was statistically different between the 2 groups (*P*=.02). Significant differences were observed in HR (*P*<.001), RR (*P*<.001), SBP (*P*<.001), DBP (*P*=.01), SpO_2_ (*P*<.001), and temperature (*P*<.001). In the eICU-CRD data set, age (*P*=.11) and ICU length of stay (*P*=.21) were not considered significant because their *P* values were greater than .05. Except for HR, which was not significant in either group (<.001 in the MIMIC-IV data set and .41 in the eICU-CRD data set), the other variables had significance levels (ie, *P*<.05; see full data in Table 1).

Next, we provided the patient characteristics for each ICU subtype, specifically general and cardiac ICUs, as shown in Multimedia Appendices 3 and 4. In the 12-hour time window for MIMIC-IV, the characteristics of general and cardiac ICUs were similar to those of the overall ICU population between the CA and non-CA groups, except for ICU length of stay (hours). In the general ICU, the length of stay was significantly longer in the CA group (*P*=.006). In the 12-hour time window for eICU-CRD, notable discrepancies were observed in the characteristics of general and cardiac ICUs between the CA and non-CA groups with respect to HR. In the general ICU, the CA group had a significantly higher HR compared with the non-CA group (*P*<.001). By contrast, the non-CA group in the cardiac ICU had a lower HR, although this difference was not statistically significant (*P*=.41).

**Table 1:** Demographic information of patients from MIMIC-IV^a^ and eICU-CRD^b^.

Characteristic		MIMIC-IV				eICU-CRD		
		Cardiac arrest (n^c^=77)	Noncardiac arrest (n=1474)	*P* value	Cardiac arrest (n=106)		Noncardiac arrest (n=3541)	*P* value
Age (year), mean (SD)		68.53 (13.63)	67.64 (13.57)	.74	60.03 (16.52)		62.65 (15.83)	.11
Intensive care unit length of stay (hours), mean (SD)		318.90 (346.82)	273.26 (142.96)	.02	199.82 (198.01)		175.28 (144.14)	.21
**Vital signs, mean (SD)**								
	Heart rate (beats/minute)	88.79 (17.60)	87.10 (17.30)	<.001	87.45 (19.54)		87.35 (17.30)	.41
	Respiratory rate (breaths/minute)	21.26 (5.68)	20.99 (5.70)	<.001	20.10 (5.90)		19.76 (4.85)	<.001
	Systolic blood pressure (mmHg)	111.21 (22.60)	118.03 (21.27)	<.001	118.88 (21.21)		125.68 (21.73)	<.001
	Diastolic blood pressure (mmHg)	59.41 (14.17)	59.64 (13.70)	.01	63.47 (14.06)		68.49 (13.86)	<.001
	Oxyhemoglobin saturation (SpO_2_)	97.22 (3.57)	96.92 (2.87)	<.001	96.94 (4.01)		96.55 (2.82)	<.001
	Temperature (°C)	36.88 (0.91)	37.12 (0.64)	<.001	36.94 (0.94)		36.90 (0.58)	<.001

^a^MIMIC: Medical Information Mart for Intensive Care.

^b^eICU-CRD: eICU-Collaborative Research Database.

^c^n: number of ICU stays.

### Feature Screening Strategy

Table 2 illustrates the efficacy of the proposed methodology, both in its original form and when combined with the ensemble feature screening process. Initially, the proposed framework was trained and validated using all features for CA prediction, with validation performed through a 10-fold LOPO cross-validation approach. The framework achieved AUROC values of 0.75, 0.99, 0.80, and 0.80 for event recall, false alarm rate, sensitivity, and specificity, respectively, on the MIMIC-IV data set. For the eICU-CRD data set, the AUROC values were 0.78, 0.99, 0.45, and 0.99, respectively, for event recall, false alarm rate, sensitivity, and specificity.

To minimize the risk of overfitting in the proposed method, feature screening was essential. An ensemble feature screening method was used to identify the optimal feature set for the best results. The selected feature sets from the ensemble screening on the MIMIC-IV data set were then incorporated into the proposed framework. This adjustment led to AUROC values of 0.79, event recall of 0.99, false alarm rate of 0.77, and sensitivity of 0.89 for the proposed framework. The selected feature sets from the ensemble feature screening on the eICU-CRD data set were incorporated into the proposed framework. This adjustment resulted in AUROC values of 0.80, event recall of 0.99, false alarm rate of 0.36, and sensitivity of 0.99. The proposed ensemble feature screening approach demonstrated superior performance compared with using all feature sets.

**Table 2:** Performance comparison without and with ensemble feature screening methods along with the proposed framework using MIMIC-IV^a^ and eICU-CRD^b^.

Method	MIMIC-IV				eICU-CRD		
	Event recall (↑^c^)	False alarm rate (↓^d^)	Sensitivity (↑)	Event recall (↑)		False alarm rate (↓)	Sensitivity (↓)
Proposed method, mean (SD)	0.99 (0.00)	0.80 (0.04)	0.80 (0.09)	0.99 (0.01)		0.45 (0.14)	0.99 (0.01)
Proposed method with feature screening, mean (SD)	0.99 (0.00)	0.77 (0.05)	0.89 (0.06)	0.99 (0.00)		0.36 (0.16)	0.99 (0.00)

^a^MIMIC: Medical Information Mart for Intensive Care.

^b^eICU-CRD: eICU-Collaborative Research Database.

^c^The ↑ symbol indicates that a higher value for the evaluation metric corresponds to a more meaningful or effective model.

^d^The ↓ symbol indicates that a lower value for the evaluation metric corresponds to more meaningful or effective model performance.

### Predictive Performance

This section presents the results of CA predictive performance. We evaluated the performance using metrics including AUROC, event recall, false alarm rate, and sensitivity.

**Figure 3:**
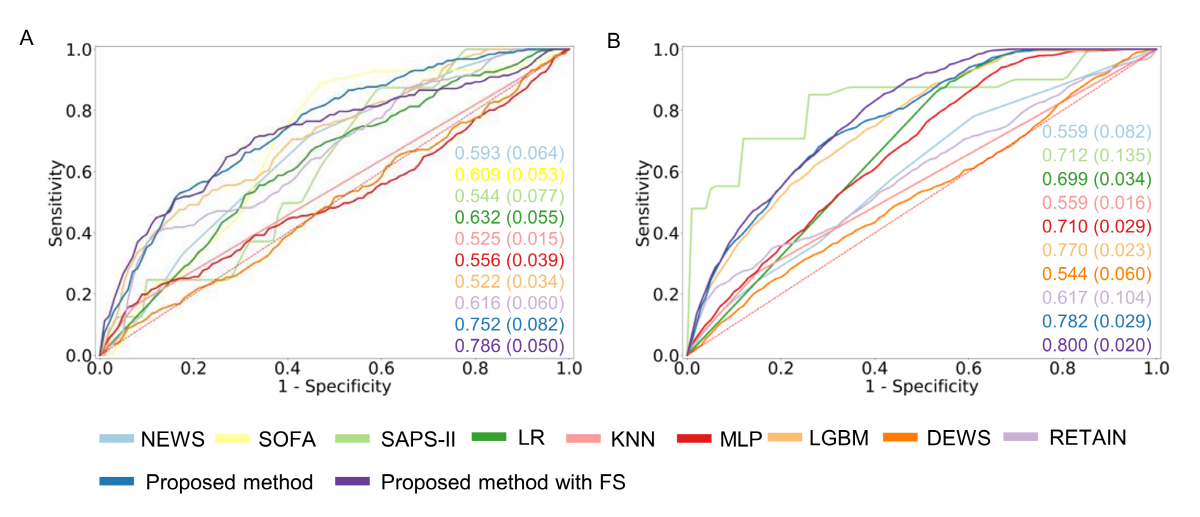
Comparison of AUROC performance among baseline models and the proposed method from MIMIC-IV and eICU-CRD. (A) AUROC from MIMIC-IV and (B) AUROC from eICU-CRD. AUROC: area under the receiver operating characteristic curve; DEWS: Deep Early Warning Score; eICU-CRD: eICU-Collaborative Research Database; FS: feature screening; KNN: K-nearest neighbors; LGBM: light gradient boosting method; LR: logistic regression; MIMIC: Medical Information Mart for Intensive Care; MLP: multilayer perceptron; NEWS: National Early Warning Score; RETAIN: reverse time attention; SAPS: Simplified Acute Physiology Score; SOFA: Sequential Organ Failure Assessment.

In the 12-hour time window from the MIMIC-IV database, we compared the AUROC of the proposed framework with that of baseline methods to investigate CA predictive performance. The proposed method achieved a higher overall AUROC value compared with the baseline methods, as shown in Figure 3. The AUROC results using the proposed method were statistically higher than those of the comparison methods (*χ*^2^_15_=68.67), as determined by the Kruskal-Wallis test with HSD post hoc analysis (see Multimedia Appendix 5). Additionally, we compared other performance metrics, including event recall, false alarm rate, and sensitivity, to assess effectiveness in a clinically relevant context. This evaluation focused on detecting CA events within a 24-hour period and minimizing false alarm rates, as shown in Table 3. The proposed method achieved statistically higher performance in event recall and sensitivity (*χ*^2^_15_=90.34 for event recall and *χ*^2^_15_=38.70 for sensitivity), as shown in Multimedia Appendices 6 and 7. Additionally, the proposed method demonstrated a statistically lower false alarm rate (*χ*^2^_15_=110.00), as detailed in Multimedia Appendix 8.

We compared the AUROC of the comparison methods and the proposed framework using the 12-hour time window from the eICU-CRD data set. The proposed method achieved statistically higher performance, with an overall AUROC value as shown in Figure 3 and Multimedia Appendix 9 (*χ*^2^_14_=81.38). Additionally, we evaluated other performance metrics, including event recall, false alarm rate, and sensitivity, as detailed in Table 4. The proposed method achieved statistically higher values for event recall and sensitivity compared with other methods, as demonstrated by the Kruskal-Wallis test with HSD (*χ*^2^_14_=90.75 for event recall, *χ*^2^_14_=100.86 for sensitivity), as shown in Multimedia Appendices 10 and 11. The proposed method also achieved a lower false alarm rate than the comparison methods, except for SAPS-II and LGBM.

**Table 3:** Comparison of LOPO^a^ cross-validation performance using MIMIC-IV^b^.

Model	Event recall (↑^c^)	False alarm rate (↓^d^)	Sensitivity (↑)
National Early Warning Score ≥5, mean (SD)	0.87 (0.12)	0.89 (0.09)	0.39 (0.10)
Sequential Organ Failure Assessment ≥6, mean (SD)	0.71 (0.13)	0.90 (0.08)	0.59 (0.15)
Simplified Acute Physiology Score-II ≥32, mean (SD)	0.91 (0.10)	0.90 (0.10)	0.91 (0.10)
Logistic regression, mean (SD)	0.99 (0.04)	0.90 (0.10)	0.69 (0.21)
k-Nearest neighbors, mean (SD)	0.05 (0.06)	0.96 (0.10)	0.01 (0.01)
Multilayer perceptron, mean (SD)	0.36 (0.13)	0.88 (0.04)	0.03 (0.02)
Light gradient boosting method, mean (SD)	0.51 (0.17)	0.87 (0.01)	0.17 (0.08)
DEWS^e^ ≥2.9, mean (SD)	0.91 (0.11)	0.92 (0.08)	0.44 (0.10)
DEWS ≥3, mean (SD)	0.91 (0.11)	0.92 (0.02)	0.44 (0.10)
DEWS ≥7.1, mean (SD)	0.85 (0.10)	0.92 (0.03)	0.37 (0.09)
DEWS ≥8, mean (SD)	0.84 (0.10)	0.92 (0.05)	0.35 (0.09)
DEWS ≥18.2, mean (SD)	0.83 (0.12)	0.92 (0.09)	0.29 (0.09)
DEWS ≥52.8, mean (SD)	0.69 (0.13)	0.92 (0.07)	0.18 (0.06)
Reverse time attention, mean (SD)	0.98 (0.05)	0.92 (0.03)	0.94 (0.10)
Proposed method, mean (SD)	0.99 (0.00)	0.80 (0.04)	0.80 (0.09)
Proposed method with feature screening, mean (SD)	0.99 (0.00)	0.77 (0.05)	0.89 (0.06)

^a^LOPO: leave-one-patient-out.

^b^MIMIC: Medical Information Mart for Intensive Care.

^c^The ↑ symbol indicates that a higher value for the evaluation metric corresponds to a more meaningful or effective model.

^d^The ↓ symbol indicates that a lower value for the evaluation metric corresponds to more meaningful or effective model performance.

^e^DEWS: Deep Learning–Based Early Warning Score.

**Table 4:** Comparison of LOPO^a^ cross-validation performance using eICU-CRD^b^.

Model	Event recall (↑^c^)	False alarm rate (↓^d^)	Sensitivity (↑)
National Early Warning Score ≥5, mean (SD)	0.99 (0.02)	0.51 (0.16)	0.68 (0.12)
Simplified Acute Physiology Score-II ≥32, mean (SD)	0.70 (0.21)	0.44 (0.19)	0.70 (0.24)
Logistic regression, mean (SD)	0.99 (0.02)	0.48 (0.14)	0.86 (0.20)
k-Nearest neighbors, mean (SD)	0.19 (0.08)	0.43 (0.27)	0.02 (0.01)
Multilayer perceptron, mean (SD)	0.54 (0.11)	0.49 (0.14)	0.08 (0.02)
Light gradient boosting method, mean (SD)	0.94 (0.05)	0.42 (0.14)	0.67 (0.05)
DEWS^e^ ≥2.9, mean (SD)	0.98 (0.03)	0.56 (0.18)	0.63 (0.09)
DEWS ≥3, mean (SD)	0.98 (0.03)	0.56 (0.18)	0.63 (0.09)
DEWS ≥7.1, mean (SD)	0.96 (0.04)	0.55 (0.18)	0.55 (0.09)
DEWS ≥8, mean (SD)	0.96 (0.04)	0.55 (0.18)	0.54 (0.09)
DEWS ≥18.2, mean (SD)	0.92 (0.06)	0.55 (0.18)	0.45 (0.08)
DEWS ≥52.8, mean (SD)	0.86 (0.09)	0.55 (0.18)	0.29 (0.06)
Reverse time attention, mean (SD)	0.99 (0.00)	0.50 (0.14)	0.99 (0.01)
Proposed method, mean (SD)	0.99 (0.01)	0.45 (0.14)	0.99 (0.01)
Proposed method with feature screening, mean (SD)	0.99 (0.00)	0.36 (0.16)	0.99 (0.01)

^a^LOPO: leave-one-patient-out.

^b^eICU-CRD: eICU-Collaborative Research Database.

^c^The ↑ symbol indicates that a higher value for the evaluation metric corresponds to a more meaningful or effective model.

^d^The ↓ symbol indicates that a lower value for the evaluation metric corresponds to more meaningful or effective model performance.

^e^DEWS: Deep Learning–Based Early Warning Score.

### Subgroup Analysis

**Figure 4:**
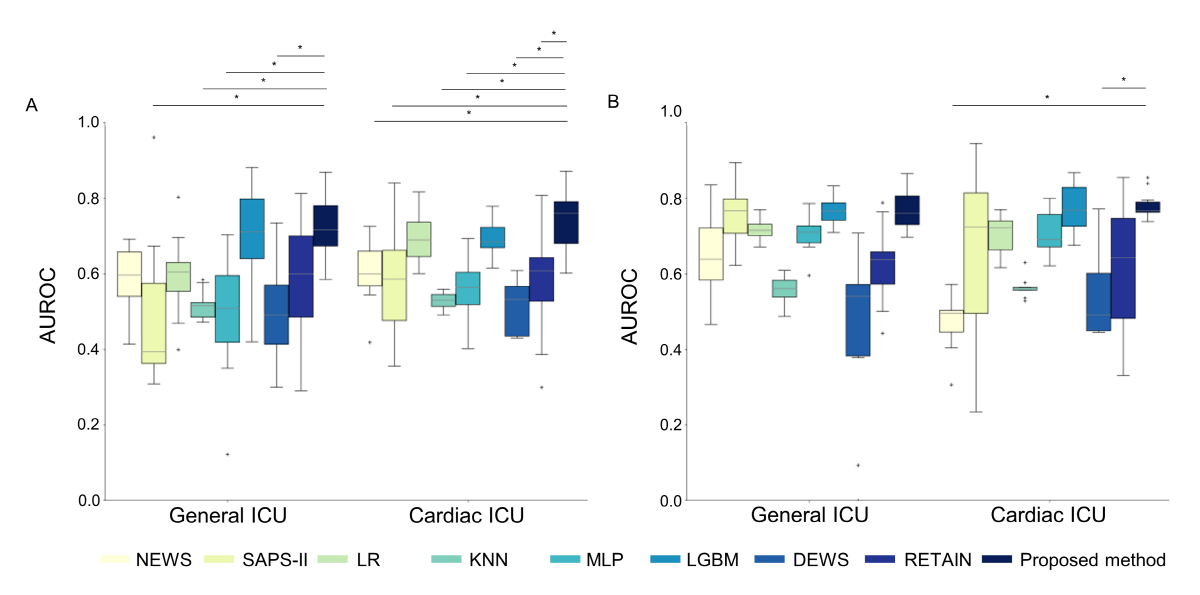
Model performance in difference patient cohorts from MIMIC-IV and eICU-CRD. (A) AUROC on ICU types of MIMIC-IV. (B) AUROC on ICU types of eICU-CRD. Boxes in the box plot show IQR and the cross marks are outliers with values that lie outside the minimum and maximum ranges of the whiskers, where minimum = Q1 - 1.5 × IQR and maximum = Q3 + 1.5 × IQR. * Statistically significant (*P*<.05). AUROC: area under the receiver operating characteristic curve; DEWS: Deep Learning–Based Early Warning Score; eICU-CRD: eICU-Collaborative Research Database; ICU: intensive care unit; KNN: k-nearest neighbors; LGBM: light gradient boosting method; LR: logistic regression; MIMIC: Medical Information Mart for Intensive Care; MLP: Multilayer perceptron; NEWS: National Early Warning Score; Q1: first quartile; Q3: third quartile; RETAIN: reverse time attention; SAPS: Simplified Acute Physiology Score.

We evaluated the performance of the comparison models and the proposed framework across different ICU types, including general and cardiac ICUs. Most ICU types showed similar performance, except for patients in the cardiac ICU within the eICU-CRD data set. As shown in Figure 4, the proposed method demonstrated statistically higher performance compared with the comparative models across all ICU types in both MIMIC-IV and eICU-CRD data sets. The comparisons by ICU type are as follows: general ICU in MIMIC-IV (*χ*^2^_8_=29.67), cardiac ICU in MIMIC-IV (*χ*^2^_8_=44.22), and cardiac ICU in eICU-CRD (*χ*^2^_8_=45.07). Detailed statistical comparison results are presented in Multimedia Appendices 12-14.

### External Validation

**Figure 5:**
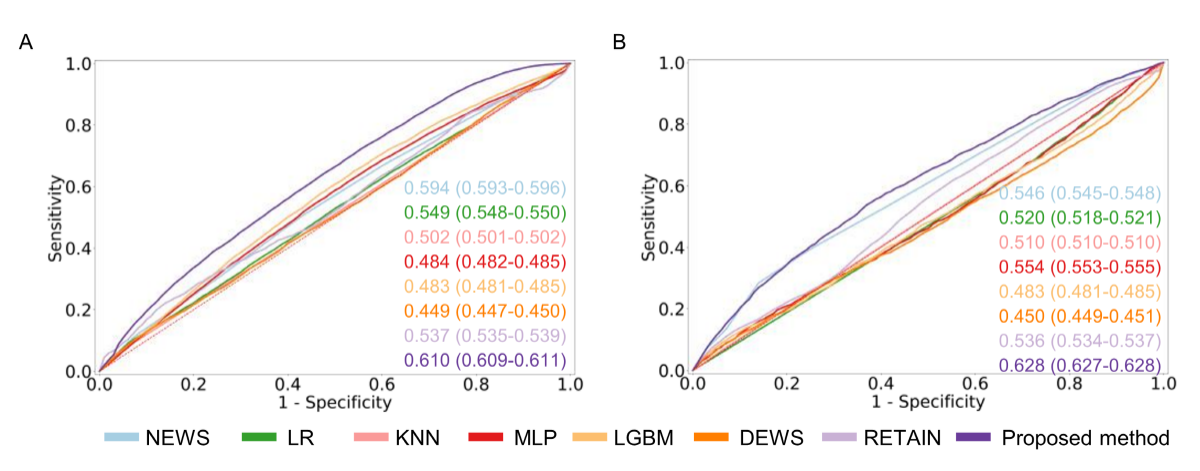
Cross–data set external validation AUROC performance. (A) eICU after training MIMIC-IV. (B) MIMIC-IV after training eICU-CRD. AUROC: area under the receiver operating characteristic curve; DEWS: Deep Learning–Based Early Warning Score; eICU-CRD: eICU-Collaborative Research Database; KNN: k-nearest neighbors; LGBM: light gradient boosting method; LR: logistic regression; MIMIC: Medical Information Mart for Intensive Care; MLP: multilayer perceptron; NEWS: National Early Warning Score; RETAIN: reverse time attention.

We conducted cross-data set external validation to assess the generalization ability of the proposed method and comparison models. After training on the MIMIC-IV data set, we evaluated the clinical validity of predicting CA within 24 hours using the eICU-CRD data set as the test set. Figure 5 and Table 5 present the external validation results for conventional systems (including NEWS, SOFA, and SAPS-II), machine learning–based comparison methods, and deep learning–based scoring systems. The proposed method achieved higher AUROC, event recall, and a lower false alarm rate compared with the comparison methods.

Conversely, we tested the proposed framework by evaluating a cohort from a general hospital setting (eICU-CRD) and a cohort with heart disease (MIMIC-IV). The results showed that the proposed framework achieved superior performance in AUROC, false alarm rate, and sensitivity, as detailed in Table 6.

**Table 5:** Cross-data set external validation performance using eICU-CRD^a^ after training MIMIC-IV^b^.

Model	Event recall (↑^c^) (95% CI)	False alarm rate (↓^d^) (95% CI)	Sensitivity (↑) (95% CI)	Brier score (95% CI)
National Early Warning Score ≥5	0.64 (0.64-0.64)	0.93 (0.93-0.93)	0.20 (0.20-0.20)	0.18 (0.18-0.18)
Logistic regression	0.74 (0.74-0.75)	0.94 (0.94-0.94)	0.25 (0.25-0.26)	0.23 (0.22-0.23)
k-Nearest neighbors	0.02 (0.02-0.02)	0.89 (0.89-0.90)	0.00 (0.00-0.00)	0.05 (0.05-0.05)
Multilayer perceptron	0.08 (0.08-0.09)	0.93 (0.93-0.93)	0.01 (0.01-0.01)	0.06 (0.06-0.06)
Light gradient boosting method	0.14 (0.14-0.14)	0.92 (0.91-0.92)	0.03 (0.03-0.03)	0.06 (0.06-0.06)
DEWS^e^ ≥2.9	0.84 (0.84-0.84)	0.97 (0.97-0.97)	0.32 (0.32-0.32)	0.09 (0.08-0.09)
DEWS ≥3	0.84 (0.84-0.84)	0.97 (0.97-0.97)	0.32 (0.31-0.32)	0.09 (0.08-0.09)
DEWS ≥7.1	0.78 (0.78-0.79)	0.97 (0.97-0.97)	0.25 (0.25-0.25)	0.09 (0.08-0.09)
DEWS ≥8	0.77 (0.77-0.77)	0.97 (0.97-0.97)	0.24 (0.24-0.24)	0.09 (0.08-0.09)
DEWS ≥18.2	0.69 (0.68-0.69)	0.97 (0.97-0.97)	0.17 (0.17-0.17)	0.08 (0.08-0.09)
DEWS ≥52.8	0.54 (0.54-0.54)	0.97 (0.97-0.97)	0.09 (0.09-0.10)	0.08 (0.08-0.09)
Reverse time attention	0.94 (0.97-0.98)	0.95 (0.95-0.95)	0.98 (0.98-0.98)	0.40 (0.40-0.40)
Proposed method	0.98 (0.98-0.99)	0.91 (0.90-0.91)	0.99 (0.99-0100)	0.30 (0.30-0.30)

^a^eICU-CRD: eICU-Collaborative Research Database.

^b^MIMIC: Medical Information Mart for Intensive Care.

^c^The ↑ symbol indicates that a higher value for the evaluation metric corresponds to a more meaningful or effective model.

^d^The ↓ symbol indicates that a lower value for the evaluation metric corresponds to more meaningful or effective model performance.

^e^DEWS: Deep Learning–Based Early Warning Score.

**Table 6:** Cross-data set external validation performance using MIMIC-IV^a^ after training eICU-CRD^b^.

Model	Event recall (↑^c^) (95% CI)	False alarm rate (↓^d^) (95% CI)	Sensitivity (↑) (95% CI)	Brier score (95% CI)
National Early Warning Score ≥5	0.99 (0.98-0.99)	0.98 (0.98-0.98)	0.84 (0.84-0.84)	0.75 (0.75-0.75)
Logistic regression	0.99 (0.98-0.99)	0.98 (0.98-0.98)	0.95 (0.95-0.95)	0.91 (0.91-0.91)
k-Nearest neighbors	0.20 (0.19-0.20)	0.98 (0.98-0.98)	0.01 (0.01-0.01)	0.04 (0.04-0.04)
Multilayer perceptron	0.69 (0.68-0.69)	0.98 (0.98-0.98)	0.10 (0.09-0.10)	0.08 (0.08-0.08)
Light gradient boosting method	0.86 (0.86-0.86)	0.98 (0.98-0.98)	0.58 (0.57-0.58)	0.40 (0.40-0.40)
DEWS^e^ ≥2.9	0.95 (0.94-0.95)	0.98 (0.98-0.98)	0.60 (0.60-0.60)	0.25 (0.25-0.25)
DEWS ≥3	0.95 (0.95-0.95)	0.98 (0.98-0.98)	0.60 (0.60-0.60)	0.25 (0.25-0.25)
DEWS ≥7.1	0.92 (0.92-0.93)	0.98 (0.98-0.98)	0.54 (0.54-0.54)	0.25 (0.25-0.25)
DEWS ≥8	0.92 (0.92-0.92)	0.98 (0.98-0.98)	0.52 (0.52-0.53)	0.25 (0.25-0.25)
DEWS ≥18.2	0.91 (0.91-0.91)	0.98 (0.98-0.98)	0.45 (0.45-0.45)	0.25 (0.25-0.25)
DEWS ≥52.8	0.83 (0.83-0.83)	0.98 (0.98-0.98)	0.29 (0.29-0.29)	0.25 (0.25-0.25)
Reverse time attention	0.99 (0.98-0.99)	0.98 (0.98-0.98)	0.99 (0.98-0.99)	0.76 (0.76-0.76)
Proposed method	0.99 (0.98-0.99)	0.50 (0.50-0.50)	0.99 (0.98-0.99)	0.31 (0.31-0.31)

^a^MIMIC: Medical Information Mart for Intensive Care.

^b^eICU-CRD: eICU-Collaborative Research Database.

^c^The ↑ symbol indicates that a higher value for the evaluation metric corresponds to a more meaningful or effective model.

^d^The ↓ symbol indicates that a lower value for the evaluation metric corresponds to more meaningful or effective model performance.

^e^DEWS: Deep Learning–Based Early Warning Score.

### Clinical Interpretability

**Figure 6:**
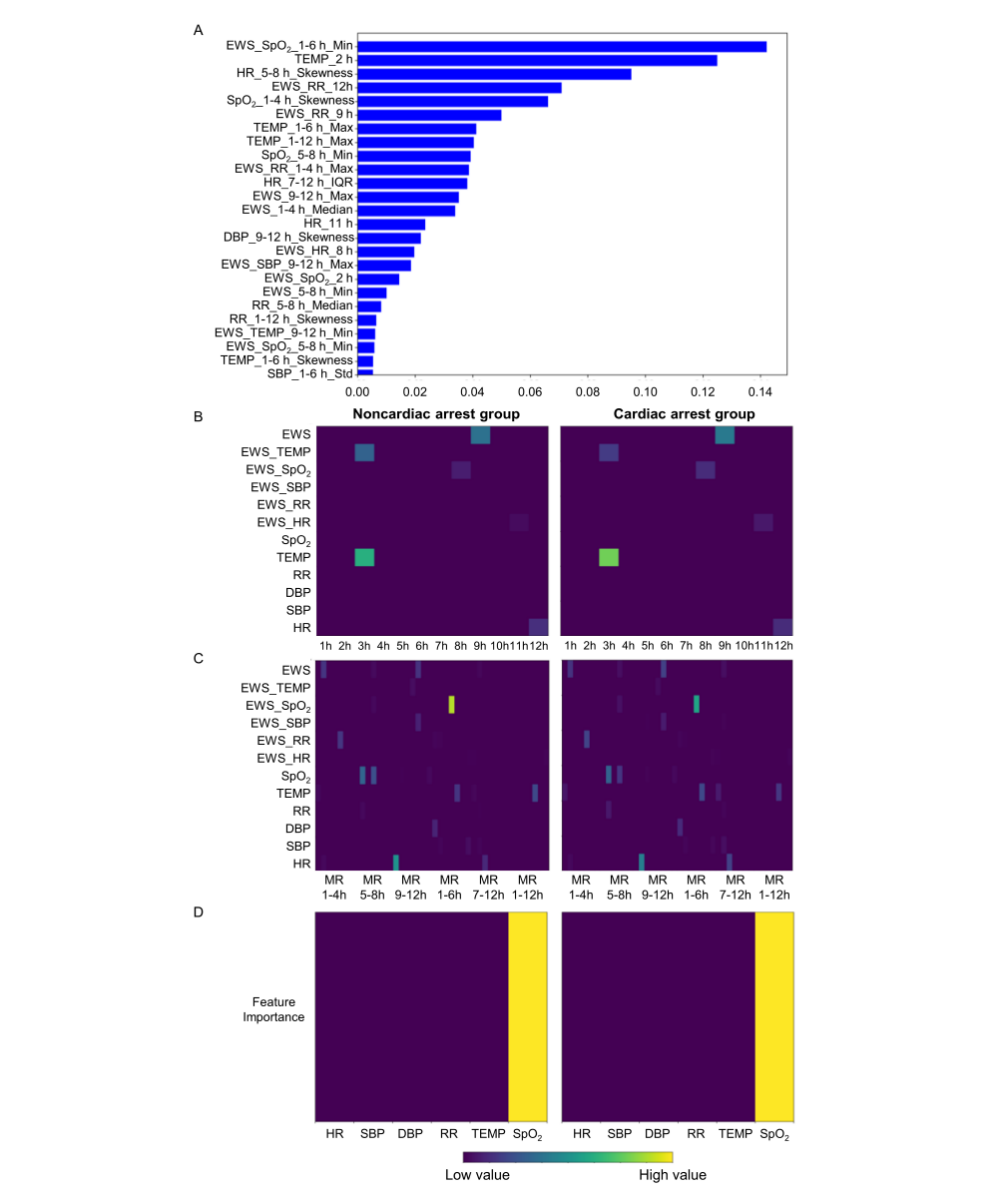
Feature inspection on MIMIC-IV. (A) Global feature impact values produced by the proposed method. (B) Vital sign–based feature set between the non-CA and CA groups. (C) Multiresolution feature set between the non-CA and CA groups. (D) Gini index–based feature set between the non-CA and CA groups. CA: cardiac arrest; DBP: diastolic blood pressure; EWS: Early warning score; HR: heart rate; Max: maximum; Min: minimum; MIMIC: Medical Information Mart for Intensive Care; MR: medical record; RR: respiratory rate; SBP: systolic blood pressure; SpO_2_: oxyhemoglobin saturation; TEMP: temperature.

We utilized local and global interpretability information from TabNet to assess the impact of each feature on the proposed model’s output. Positive importance values indicated features that increased prediction scores, while negative values indicated features that decreased prediction scores. Figure 6 displays the top 25 features of the proposed model.

Specifically, the features EWS_SpO_2__1_6 h_Min (*P*<.001), Temp_2 h (*P*<.001), and HR_5_8 h_Skewness (*P*<.001) had relatively significant impacts on the performance of the proposed method, as shown in Figure 6A. Additionally, most of the top 25 influential features were based on vital signs and multiresolution statistical features. Of these, 20 (all features except DBP_9_12h_Skewness, EWS_HR_8h, and EWS_SBP_9_12h_Max: *P*<.001; DBP_9_12h_Skewness: *P=*.02; EWS_HR_8h: *P=*.03; and EWS_SBP_9_12h_Max: *P=*.01) out of the 25 global interpretability features showed statistically significant differences between the non-CA and CA groups, as determined by an independent *t* test with FDR correction, detailed in Multimedia Appendix 15. In the multiresolution statistical features based on the sliding window, temperature (all *P*<.001), EWS-SpO_2_ (all *P*<.001), EWS-RR (all *P*<.001), EWS-total (EWS_9_12 h_max: *P<*.001), EWS-SBP (all *P*<.001), HR (all *P*<.001), TEMP (all *P*<.001), SpO_2_ (SpO_2__5_8 h_Min: *P*<.001), and DBP (*P*=.02) had statistically significant effects on the proposed model, as shown in Figure 6C [47]. Additionally, the Gini index–based function, calculated over a 12-hour window for SpO_2_, emerged as the most important indicator for both the non-CA and CA groups, as shown in Figure 6D [48].

### Computational Cost Analysis

We analyzed the computational complexity and time required for model training and prediction of both the comparison models and the proposed framework to assess their applicability in real-time critical care settings. Computational complexity measures the amount of computing resources consumed by an algorithm [49] and can be expressed in terms of both time and space. In essence, computational complexity measures how quickly or slowly an algorithm performs relative to a given input size. It is specified in terms of time complexity and space complexity. Time complexity refers to the amount of computational time an algorithm requires, while space complexity describes the amount of additional memory needed to execute the algorithm. Both types of complexity are expressed relative to a specific input size, which includes factors such as the number of training examples, the number of features, the depth and number of trees, and the length and dimension of the input.

Table 7 illustrates the computational complexity of both the comparative models and the proposed framework, categorized by time and space complexity. Additionally, the table depicts the actual training time and the time required to generate results with the trained model, expressed as training and inference time, respectively. As a result, the proposed method required 31.82 and 60.51 seconds of training time per epoch on the MIMIC-IV and eICU-CRD data sets, respectively. Additionally, the prediction time for the proposed method was 0.25 and 0.54 seconds on the MIMIC-IV and eICU-CRD data sets, respectively. In comparison, LGBM demonstrated the shortest training time and fastest prediction time, making it the most lightweight algorithm. The proposed method required the least training and prediction time among the deep learning–based methods. Although the training time was longer than that of LGBM, the prediction time was not significantly different from that of LGBM (MIMIC-IV: *P*=.11, eICU-CRD: *P*=.08).

**Table 7:** Comparisons of computational complexity and actual training and inference time.

Method	Computational complexity		MIMIC-IV^a^		eICU-CRD^b^	
	Time	Space	Time per training (seconds)	Inference time (seconds)	Time per training (seconds)	Inference time (seconds)
National Early Warning Score	—^c^	—	—	—	—	—
Simplified Acute Physiology Score-II	—	—	—	—	—	—
Logistic regression	*O*(*n*^d^*f*^e^)	O(*f*)	27.69	0.02	52.62	0.03
k-Nearest neighbors	*O*[*fn*log(*n*)]	*O*(*nf*)	0.04	33.18	0.08	136.24
Multilayer perceptron	*O*(*nfl*^2^_oi_)	—	624.98	2.51	5061.21	6.48
Light gradient boosting method	*O*[*n*log(*n*)]*k*^f^	*O*[*n*log(*n*)]	4.94	0.06	7.17	0.09
Deep Learning–Based Early Warning Score	*O*(*N*^g^*D*^2h^)	*O*(*ND*^2^)	1384.37	0.87	2907.94	1.33
Reverse time attention	*O*(*ND*^2^)	*O*(*ND*^2^)	1497.30	0.06	2906.91	0.10
Proposed method	*O*(*ND*^2^)	*O*(*ND*^2^)	31.82	0.25	60.51	0.54

^a^MIMIC: Medical Information Mart for Intensive Care.

^b^eICU-CRD: eICU-Collaborative Research Database.

^c^Not available.

^d^n: number of training examples.

^e^f: number of features.

^f^k: number of trees.

^g^N: the length of the input.

^h^D: the dimension of the input.

## Discussion

### Principal Findings

Clinicians can use the proposed model to make consistent clinical decisions for patients in various ICUs with similar organ failure types, facilitating efficient management [50]. This study developed and validated an ensemble-based model capable of predicting CA events 24 hours in advance, regardless of different hospitalization settings, patient populations, and ICU subtypes within each database, as well as across external validations. Therefore, the proposed method can help reduce the number of CA events and ICU deaths without compromising performance due to heterogeneity. Additionally, it lowers the false alarm rate compared with existing methods, providing clinicians with more reliable CA event alarms and better preparedness in hospitals.

For CA prediction across different patient populations, the proposed method, utilizing 3 types of features, demonstrated statistically superior performance in AUROC, sensitivity, false alarm rate, and event recall compared with the comparison models. The proposed method achieved significant performance improvements in predicting CA events for the entire ICU patient group (AUROC: *P*<.001; sensitivity: *P*<.001; and event recall: *P*<.001) and showed even greater statistical differences in high-risk patients with CA (AUROC: *P*<.001; sensitivity: *P*<.001; false alarm rate: *P*<.001; and event recall: *P*<.001). Therefore, the proposed method is crucial for predicting significant CA events in both the high-risk CA group and the entire ICU population.

We evaluated the performance of both the comparison models and the proposed framework across different ICU types, including general and cardiac ICUs. We found similar performance levels across most ICU types, except for patients in the cardiac ICU of the eICU-CRD.

For the MIMIC-IV data set, which includes patients with cardiac-related diseases in the ICU, the class distribution rate between the CA and non-CA groups is 3.2% (24/738; CA group, n=24 and non-CA group, n=738) in the general ICU. In the cardiac ICU, the ratio is 5.8% (43/741; CA group, n=43 and non-CA group, n=741), which is slightly higher than in the general ICU. The AUROC performance improved in all cases except for LGBM in the cardiac ICU, where the distribution trend increased, although LGBM’s performance was similar between the general and cardiac ICUs. Overall, the AUROC performance for each ICU type, including both general and cardiac ICUs, was consistent with the performance observed across all patients. The AUROC performance of NEWS and SAPS-II in the cardiac ICU improved by 0.02 and 0.10, respectively, compared with their performance in the general ICU. This finding aligns with the analysis results in MIMIC-IV, which indicated that vital sign–based features had a strong influence on performance, as mentioned in the results of the analysis of the effect of the feature set on performance in MIMIC-IV.

When analyzing the AUROC for each ICU type in the eICU-CRD, which includes all hospitalized ICU patients, the performance patterns differed from those observed in the MIMIC-IV data set. The class distribution ratio between the CA and non-CA groups in both the general and cardiac ICUs in the eICU-CRD is similar to that in MIMIC-IV. Specifically, in the general ICU of eICU-CRD, the class distribution rate between the CA and non-CA groups is 2.35% (60/2554; CA group, n=60 and non-CA group, n=2554). In the cardiac ICU, the ratio is 5.80% (43/741; CA group, n=43 and non-CA group, n=741). However, the AUROC performance of NEWS and SAPS-II decreased by 0.17 and 0.10, respectively. Additionally, the AUROC performance of the other comparison models remained similar, despite the increased class ratio of the CA group in the cardiac ICU of the eICU-CRD.

### Strengths

This study has several strengths. First, we assessed the performance of predicting CA across different patient populations and hospital settings. Moreover, we evaluated the predictive performance of CA over the entire hospitalization period for each patient, rather than relying on event-based assessments that represent the patient’s CA or normal condition. Second, we optimized the hyperparameter values of each machine learning model through iterative grid search. Hyperparameter tuning has been shown to improve the performance of these models. We proposed an interpretable and calibrated ensemble approach using TabNet with different cost weights for each class to predict CA events within 24 hours. The proposed feature sets, defined according to the AI development cycle, and TabNet demonstrated significantly higher CA prediction accuracy compared with the baseline models (all *P*<.001). Therefore, clinicians would have sufficient time to respond to CA events when using the proposed model. Third, we examined the extent to which bias in the data influenced our framework. Ultimately, our proposed framework outperformed all comparative models when trained and tested on these diverse populations. This demonstrates that the framework has strong generalization capabilities and is not overly affected by the confounding effects of specific group characteristics in the learning data.

We analyzed the computational complexity and time required for model training and prediction to determine whether the comparative models and the proposed framework could be applied in real-time within the critical care field. Although the training time for the proposed method was longer than that for LGBM, the prediction time was comparable. Therefore, the proposed method can deliver prediction results without delay in response time, making it suitable for clinical use in critical care settings and integration into existing systems.

### Limitations

This study has several limitations. Although the proposed model demonstrated superior performance in external validation, further validation using independent data sets is necessary. Additionally, the ensemble approach based on TabNet was developed without feature screening. However, as the high-performance results indicate, this did not greatly impact the model’s effectiveness. Although the proposed method demonstrated higher precision, sensitivity, specificity, AUROC, and a lower false alarm rate, the false alarm rate remains high, which is a limitation of our study. Further research is needed to develop feature-generation methods and models that can further reduce the false alarm rate. In the future, the proposed model could be optimized to include feature screening. Nevertheless, as discussed, the prediction model in this study shows strong potential for clinical application in CDSSs and early interventions. Accessibility and user experience can be enhanced by implementing a user-centered CDSS or web-based application according to the proposed model. Lastly, although we extensively validated the framework on various data sets and subgroups through retrospective analysis, achieving clinical maturity will require real-time prospective studies. Therefore, further research is necessary to ensure clinical maturity through prospective clinical studies in critical care environments.

### Conclusions

In this study, we evaluated the performance of an interpretable AI alert model across different ICU populations in a simulated real-time clinical setting. As these results were tested in real-time clinical scenarios based on various databases for each patient group, we anticipate that similar outcomes will be observed in actual clinical trials. Consequently, the test results are expected to be valuable and applicable in real clinical settings. We then used the interpretable information from the proposed method to describe both global and time-specific relevance. This clinical descriptive information aids clinicians in decision-making by highlighting the associations between predicted outcomes and patient characteristics. Consequently, our CA prediction system is considered to have achieved clinical validity and is now being used and validated for routine clinical applications.

## Appendix

Multimedia Appendix 1 Source code information.

Multimedia Appendix 2 Details about the hyperparameters of the baseline models.

Multimedia Appendix 3 Demographic information of patients from subgroups of MIMIC-IV. MIMIC: Medical Information Mart for Intensive Care.

Multimedia Appendix 4 Demographic information of patients from subgroups of eICU-CRD. eICU-CRD: eICU-Collaborative Research Database.

Multimedia Appendix 5 Statistical comparison of the overall area under the receiver operating characteristic curve between the proposed method and baseline methods on the MIMIC-IV data set. MIMIC: Medical Information Mart for Intensive Care.

Multimedia Appendix 6 Statistical comparison of the overall event recall between the proposed method and baseline methods on the MIMIC-IV. MIMIC: Medical Information Mart for Intensive Care.

Multimedia Appendix 7 Statistical comparison of the overall sensitivity between the proposed method and baseline methods on the MIMIC-IV. MIMIC: Medical Information Mart for Intensive Care.

Multimedia Appendix 8 Statistical comparison of the overall false alarm rate between the proposed method and baseline methods on the MIMIC-IV. MIMIC: Medical Information Mart for Intensive Care.

Multimedia Appendix 9 Statistical comparison of the overall area under the receiver operating characteristic curve between the proposed method and baseline methods on the eICU-CRD. eICU-CRD: eICU-Collaborative Research Database.

Multimedia Appendix 10 Statistical comparison of the overall event recall between the proposed method and baseline methods on the eICU-CRD. eICU-CRD: eICU-Collaborative Research Database.

Multimedia Appendix 11 Statistical comparison of the overall sensitivity between the proposed method and baseline methods on the eICU-CRD. eICU-CRD: eICU-Collaborative Research Database.

Multimedia Appendix 12 Statistical comparison of the overall area under the receiver operating characteristic curve between the proposed method and baseline methods on the MIMIC-IV in cardiac ICU. ICU: intensive care unit; MIMIC: Medical Information Mart for Intensive Care.

Multimedia Appendix 13 Statistical comparison of the overall area under the receiver operating characteristic curve between the proposed method and baseline methods on the MIMIC-IV in general ICU. ICU: intensive care unit; MIMIC: Medical Information Mart for Intensive Care.

Multimedia Appendix 14 Statistical comparison of the overall area under the receiver operating characteristic curve between the proposed method and baseline methods on the eICU-CRD in cardiac ICU. eICU-CRD: eICU-Collaborative Research Database; ICU: intensive care unit.

Multimedia Appendix 15 Statistical comparison between Gini index–based feature of SpO_2_ and the top 25 global importance features in the proposed method. SpO_2_: oxyhemoglobin saturation.

## Abbreviations

AI: artificial intelligence
AUPRC: area under the precision-recall curve
AUROC: area under the receiver operating characteristic curve
BIDMC: Beth Israel Deaconess Medical Center
CA: cardiac arrest
CDSS: clinical decision support system
DBP: diastolic blood pressure
eICU-CRD: eICU-Collaborative Research Database
EWS: early warning score
FDR: false discovery rate
HF: heart failure
HR: heart rate
HSD: honestly significant difference
ICU: intensive care unit
LGBM: light gradient boosting method
LOCB: last observation carried backward
LOCF: last observation carried forward
LOPO: leave-one-patient-out
MEWS: Modified Early Warning Score
MIMIC: Medical Information Mart for Intensive Care
NEWS: National Early Warning Score
RF: random forest
RNN: recurrent neural network
RR: respiratory rate
SAPS: Simplified Acute Physiology Score
SBP: systolic blood pressure
SOFA: Sequential Organ Failure Assessment
SpO_2_: oxyhemoglobin saturation
TabNet: tabular network

## Data Availability

MIMIC-IV is available online upon request at the repository PhysioNet [51]. The eICU-CRD is also freely available through PhysioNet to access [52]. The source code generated and used for this study is publicly available for download in Multimedia Appendix 1. The source code for Scikit-learn of Python is freely available at [53]. The source code for TabNet is freely available at [54].

## References

[1] Nolan JP, Berg RA, Andersen LW, Bhanji F, Chan PS, Donnino MW, Lim SH, Ma MH, Nadkarni VM, Starks MA, Perkins GD, Morley PT, Soar J (2019). Cardiac arrest and cardiopulmonary resuscitation outcome reports: update of the Utstein Resuscitation Registry Template for in-hospital cardiac arrest: a consensus report from a Task Force of the International Liaison Committee on Resuscitation (American Heart Association, European Resuscitation Council, Australian and New Zealand Council on Resuscitation, Heart and Stroke Foundation of Canada, InterAmerican Heart Foundation, Resuscitation Council of Southern Africa, Resuscitation Council of Asia). Circulation.

[2] Andersen LW, Kim WY, Chase M, Berg KM, Mortensen SJ, Moskowitz A, Novack V, Cocchi MN, Donnino MW, American Heart Association's Get With the Guidelines(®) – Resuscitation Investigators (2016). The prevalence and significance of abnormal vital signs prior to in-hospital cardiac arrest. Resuscitation.

[3] Bergum D, Haugen BO, Nordseth T, Mjølstad Ole Christian, Skogvoll E (2015). Recognizing the causes of in-hospital cardiac arrest--a survival benefit. Resuscitation.

[4] Guidi G, Pettenati MC, Melillo P, Iadanza E (2014). A machine learning system to improve heart failure patient assistance. IEEE J Biomed Health Inform.

[5] Jeong J, Cho J, Lee B, Lee S, Jeong J (2023). Real-time deep neurolinguistic learning enhances noninvasive neural language decoding for brain–machine interaction. IEEE Trans Cybern.

[6] Sutton RT, Pincock D, Baumgart DC, Sadowski DC, Fedorak RN, Kroeker KI (2020). An overview of clinical decision support systems: benefits, risks, and strategies for success. NPJ Digit Med.

[7] Zhou C, Li A, Hou A, Zhang Z, Zhang Z, Dai P, Wang F (2020). Modeling methodology for early warning of chronic heart failure based on real medical big data. Expert Syst Appl.

[8] Jain A, Chandra Sekhara Rao A, Kumar Jain P, Hu Y (2023). Optimized levy flight model for heart disease prediction using CNN framework in big data application. Expert Syst Appl.

[9] Alsalem M, Alamoodi A, Albahri O, Albahri A, Martínez L, Yera R, Duhaim AM, Sharaf IM (2024). Evaluation of trustworthy artificial intelligent healthcare applications using multi-criteria decision-making approach. Expert Syst Appl.

[10] Sidek KA, Khalil I, Jelinek HF (2014). ECG biometric with abnormal cardiac conditions in remote monitoring system. IEEE Trans Syst Man Cybern Syst.

[11] Aegerter P, Boumendil A, Retbi A, Minvielle E, Dervaux B, Guidet B (2005). SAPS-II revisited. Intensive Care Med.

[12] Spångfors Martin, Molt M, Samuelson K (2020). In-hospital cardiac arrest and preceding National Early Warning Score (NEWS): a retrospective case-control study. Clin Med (Lond).

[13] Yijing L, Wenyu Y, Kang Y, Shengyu Z, Xianliang H, Xingliang J, Cheng W, Zehui S, Mengxing L (2022). Prediction of cardiac arrest in critically ill patients based on bedside vital signs monitoring. Comput Methods Programs Biomed.

[14] Vincent J, Moreno R, Takala J, Willatts S, Mendonça AD, Bruining H, Reinhart CK, Suter PM, Thijs LG (1996). The SOFA (Sepsis-related Organ Failure Assessment) score to describe organ dysfunction/failure. Intensive Care Med.

[15] Subbe C, Kruger M, Rutherford P, Gemmel L (2001). Validation of a modified Early Warning Score in medical admissions. QJM.

[16] Smith GB, Prytherch DR, Schmidt PE, Featherstone PI, Higgins B (2008). A review, and performance evaluation, of single-parameter "track and trigger" systems. Resuscitation.

[17] Kwon J, Lee Y, Lee Y, Lee S, Park J (2018). An algorithm based on deep learning for predicting in‐hospital cardiac arrest. J Am Heart Assoc.

[18] Kim YK, Koo JH, Lee SJ, Song HS, Lee M (2023). Explainable artificial intelligence warning model using an ensemble approach for in-hospital cardiac arrest prediction: retrospective cohort study. J Med Internet Res.

[19] Churpek MM, Yuen TC, Winslow C, Meltzer DO, Kattan MW, Edelson DP (2016). Multicenter comparison of machine learning methods and conventional regression for predicting clinical deterioration on the wards. Critical Care Med.

[20] Hong S, Lee S, Lee J, Cha WC, Kim K (2020). Prediction of cardiac arrest in the emergency department based on machine learning and sequential characteristics: model development and retrospective clinical validation study. JMIR Med Inform.

[21] Layeghian Javan S, Sepehri MM, Layeghian Javan M, Khatibi T (2019). An intelligent warning model for early prediction of cardiac arrest in sepsis patients. Comput Methods Programs Biomed.

[22] Sharma M, Savage C, Nair M, Larsson I, Svedberg P, Nygren JM (2022). Artificial Intelligence Applications in Health Care Practice: Scoping Review. J Med Internet Res.

[23] Luo W, Phung D, Tran T, Gupta S, Rana S, Karmakar C, Shilton A, Yearwood J, Dimitrova N, Ho TB, Venkatesh S, Berk M (2016). Guidelines for developing and reporting machine learning predictive models in biomedical research: a multidisciplinary view. J Med Internet Res.

[24] Arik SÖ, Pfister T (2021). TabNet: attentive interpretable tabular learning.

[25] Johnson A, Bulgarelli L, Pollard T, Horng S, Celi L, Mark R MIMIC-IV (version 1). PhysioNet.

[26] Pollard TJ, Johnson AEW, Raffa JD, Celi LA, Mark RG, Badawi O (2018). The eICU Collaborative Research Database, a freely available multi-center database for critical care research. Sci Data.

[27] Rajkomar A, Hardt M, Howell MD, Corrado G, Chin MH (2018). Ensuring fairness in machine learning to advance health equity. Ann Intern Med.

[28] Gebru T, Morgenstern J, Vecchione B, Vaughan JW, Wallach H, III HD, Crawford K (2021). Datasheets for datasets. Commun ACM.

[29] Haibe-Kains B, Adam GA, Hosny A, Khodakarami F, Waldron Levi, Wang Bo, McIntosh Chris, Goldenberg Anna, Kundaje Anshul, Greene Casey S, Broderick Tamara, Hoffman Michael M, Leek Jeffrey T, Korthauer Keegan, Huber Wolfgang, Brazma Alvis, Pineau Joelle, Tibshirani Robert, Hastie Trevor, Ioannidis John P A, Quackenbush John, Aerts Hugo J W L, Massive Analysis Quality Control (MAQC) Society Board of Directors (2020). Transparency and reproducibility in artificial intelligence. Nature.

[30] Ueda D, Kakinuma T, Fujita S, Kamagata K, Fushimi Y, Ito R, Matsui Y, Nozaki T, Nakaura T, Fujima N, Tatsugami F, Yanagawa M, Hirata K, Yamada A, Tsuboyama T, Kawamura M, Fujioka T, Naganawa S (2024). Fairness of artificial intelligence in healthcare: review and recommendations. Jpn J Radiol.

[31] Marmot M, Bell R (2012). Fair society, healthy lives. Public Health.

[32] Anderson M, Anderson S (2019). How should AI be developed, validated, and implemented in patient care?. AMA J Ethics.

[33] Walsh C, Chaudhry B, Dua P, Goodman K, Kaplan B, Kavuluru R, Solomonides Anthony, Subbian Vignesh (2020). Stigma, biomarkers, and algorithmic bias: recommendations for precision behavioral health with artificial intelligence. JAMIA Open.

[34] GitHub.

[35] Barnard J, Meng X (1999). Applications of multiple imputation in medical studies: from AIDS to NHANES. Stat Methods Med Res.

[36] Kenward MG, Molenberghs G (2009). Last observation carried forward: a crystal ball?. J Biopharm Stat.

[37] Goodwin T, Demner-Fushman D (2020). A customizable deep learning model for nosocomial risk prediction from critical care notes with indirect supervision. J Am Med Inform Assoc.

[38] Darst BF, Malecki KC, Engelman CD (2018). Using recursive feature elimination in random forest to account for correlated variables in high dimensional data. BMC Genet.

[39] Manikandan G, Pragadeesh B, Manojkumar V, Karthikeyan A, Manikandan R, Gandomi AH (2024). Classification models combined with Boruta feature selection for heart disease prediction. Inform Med Unlocked.

[40] Kotsiantis S, Pintelas P (2003). Mixture of expert agents for handling imbalanced data sets. Ann Math Comput Teleinform.

[41] Li F, Zhang X, Zhang X, Du C, Xu Y, Tian Y (2018). Cost-sensitive and hybrid-attribute measure multi-decision tree over imbalanced data sets. Inform Sci.

[42] Naseem U, Rashid J, Ali L, Kim J, Haq QEU, Awan MJ, Imran M (2022). An automatic detection of breast cancer diagnosis and prognosis based on machine learning using ensemble of classifiers. IEEE Access.

[43] Gholamiangonabadi D, Kiselov N, Grolinger K (2020). Deep neural networks for human activity recognition with wearable sensors: leave-one-subject-out cross-validation for model selection. IEEE Access.

[44] Pauli M, Pohl C, Golz M (2021). Balanced leave-one-subject-out cross-validation for microsleep classification. Curr Dir Biomed Eng.

[45] Newman-Griffis D, Divita G, Desmet B, Zirikly A, Rosé Carolyn P, Fosler-Lussier E (2021). Ambiguity in medical concept normalization: an analysis of types and coverage in electronic health record datasets. J Am Med Inform Assoc.

[46] Graham R, Margaret AM, Andrea MS (2015). Strategies to Improve Cardiac Arrest Survival: A Time to Act.

[47] Hyland SL, Faltys M, Hüser Matthias, Lyu X, Gumbsch T, Esteban C, Bock C, Horn M, Moor M, Rieck B, Zimmermann M, Bodenham D, Borgwardt K, Rätsch Gunnar, Merz TM (2020). Early prediction of circulatory failure in the intensive care unit using machine learning. Nat Med.

[48] Lowry A, Futterman C, Gazit A (2022). Acute vital signs changes are underrepresented by a conventional electronic health record when compared with automatically acquired data in a single-center tertiary pediatric cardiac intensive care unit. J Am Med Inform Assoc.

[49] Papadimitriou C (2003). Computational complexity. Ency Comput Sci.

[50] Rossetti S, Knaplund C, Albers D, Dykes Patricia C, Kang Min Jeoung, Korach Tom Z, Zhou Li, Schnock Kumiko, Garcia Jose, Schwartz Jessica, Fu Li-Heng, Klann Jeffrey G, Lowenthal Graham, Cato Kenrick (2021). Healthcare process modeling to phenotype clinician behaviors for exploiting the signal gain of clinical expertise (HPM-ExpertSignals): development and evaluation of a conceptual framework. J Am Med Inform Assoc.

[51] PhysioNet.

[52] eICU Collaborative Research Database - MIT.

[53] Scikit-learn.

[54] GitHub.

