# Correction: Early Prediction of Cardiac Arrest in the Intensive Care Unit Using Explainable Machine Learning: Retrospective Study

**DOI:** 10.2196/67135

**Published:** 2024-10-28

**Authors:** Yun Kwan Kim, Won-Doo Seo, Sun Jung Lee, Ja Hyung Koo, Gyung Chul Kim, Hee Seok Song, Minji Lee

**Affiliations:** 1 Technology Development Seers Technology Co. Ltd. Pyeongtaek-si, Gyeonggi-do Republic of Korea; 2 Department of Brain and Cognitive Engineering Korea University Seoul Republic of Korea; 3 Department of Biomedical Software Engineering The Catholic University of Korea Bucheon-si, Gyeonggi-do Republic of Korea

In “Early Prediction of Cardiac Arrest in the Intensive Care Unit Using Explainable Machine Learning: Retrospective Study” (J Med Internet Res 2024;26:e62890) the authors noted one error:

In "Results", "Subgroup Analysis", Figure 4 has been replaced with Figure 5, as follows: 

**Figure 4:**
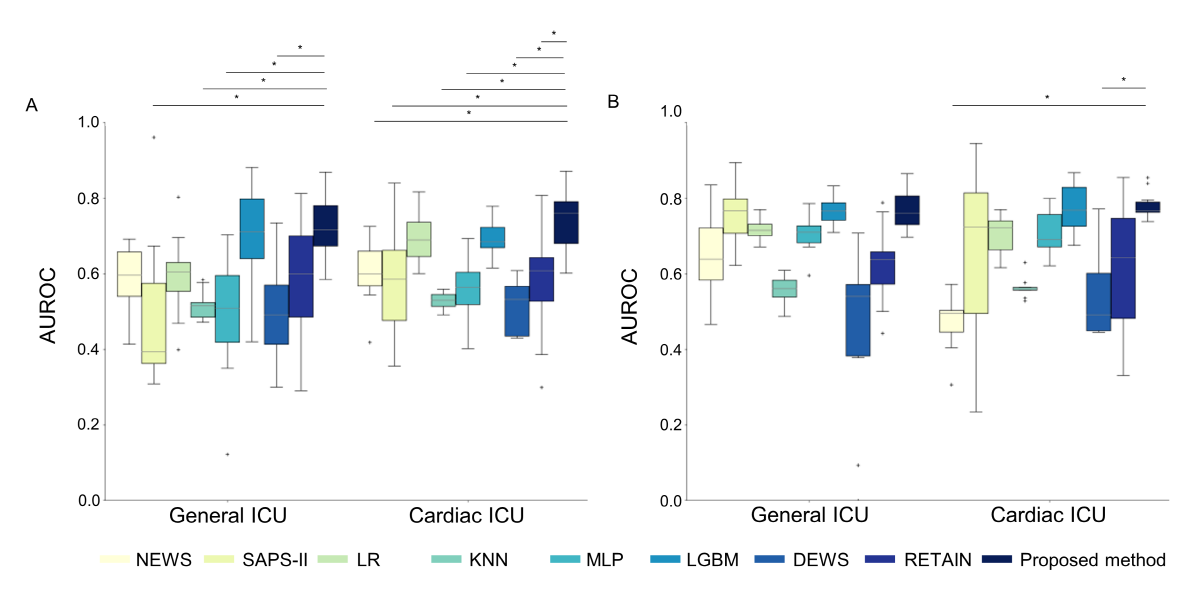
Model performance in difference patient cohorts from MIMIC-IV and eICU-CRD. (A) AUROC on ICU types of MIMIC-IV. (B) AUROC on ICU types of eICU-CRD. Boxes in the box plot show IQR and the cross marks are outliers with values that lie outside the minimum and maximum ranges of the whiskers, where minimum = Q1 - 1.5 × IQR and maximum = Q3 + 1.5 × IQR. * Statistically significant (*P*<.05). AUROC: area under the receiver operating characteristic curve; DEWS: Deep Learning–Based Early Warning Score; eICU-CRD: eICU-Collaborative Research Database; ICU: intensive care unit; KNN: k-nearest neighbors; LGBM: light gradient boosting method; LR: logistic regression; MIMIC: Medical Information Mart for Intensive Care; MLP: Multilayer perceptron; NEWS: National Early Warning Score; Q1: first quartile; Q3: third quartile; RETAIN: reverse time attention; SAPS: Simplified Acute Physiology Score.

In "Results", "External Validation", Figure 5 has been replaced with Figure 4, as follows: 

**Figure 5:**
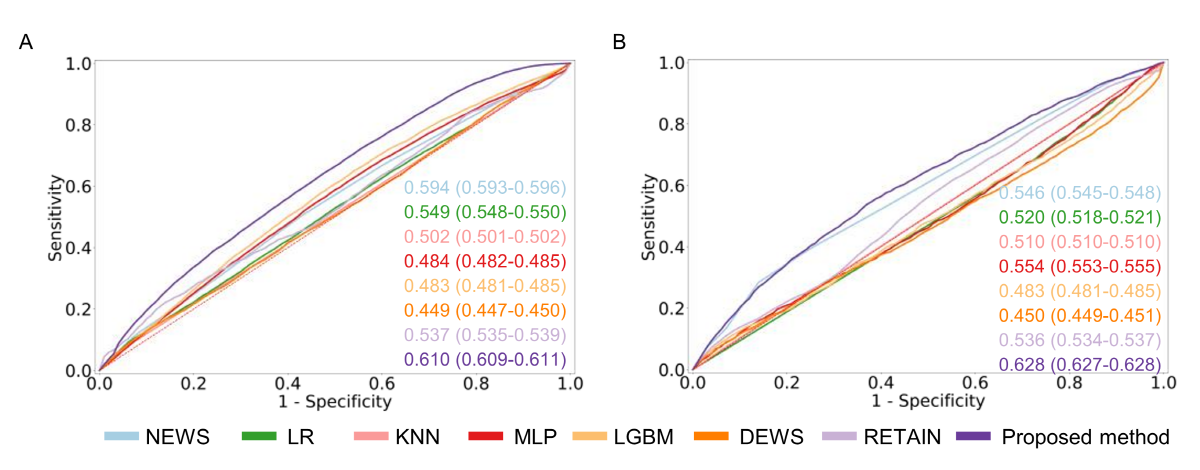
Cross–data set external validation AUROC performance. (A) eICU after training MIMIC-IV. (B) MIMIC-IV after training eICU-CRD. AUROC: area under the receiver operating characteristic curve; DEWS: Deep Learning–Based Early Warning Score; eICU-CRD: eICU-Collaborative Research Database; KNN: k-nearest neighbors; LGBM: light gradient boosting method; LR: logistic regression; MIMIC: Medical Information Mart for Intensive Care; MLP: multilayer perceptron; NEWS: National Early Warning Score; RETAIN: reverse time attention.

The correction will appear in the online version of the paper on the JMIR Publications website on October 28, 2024 together with the publication of this correction notice. Because this was made after submission to PubMed, PubMed Central, and other full-text repositories, the corrected article has also been resubmitted to those repositories.

