# Supplementary material for: Early Prediction of Cardiac Arrest in the Intensive Care Unit Using Explainable Machine Learning: Retrospective Study
Source: J Med Internet Res. 2024 Sep 17;26:e62890. doi: 10.2196/62890 (PMC11445627; doi:10.2196/62890)
Supplement: Multimedia Appendix 1 [file jmir_v26i1e62890_app1.docx]

**Multimedia Appendix 1.** Source code information.

Source code generated and used for this study is publicly available for download at https://github.com/yunkwankim/SCAP. Source code for scikit-learn packages of python is freely available at https://scikit-learn.org/stable/. Source code for TabNet is freely available at https://github.com/topics/pytorch-tabnet.
