# Supplementary material for: Early Prediction of Cardiac Arrest in the Intensive Care Unit Using Explainable Machine Learning: Retrospective Study
Source: J Med Internet Res. 2024 Sep 17;26:e62890. doi: 10.2196/62890 (PMC11445627; doi:10.2196/62890)
Supplement: Multimedia Appendix 2 [file jmir_v26i1e62890_app2.docx]

**Multimedia Appendix 2.** Details about the Hyperparameters of the Baseline Models.

| **Model** | **Hyperparameter** | **Class Weight**  **(Cardiac Arrest Group)** |
| --- | --- | --- |
| **NEWS**^a^ | Cut-off threshold of MIMIC-IV = 5  Cut-off threshold of eICU-CRD = 6 | - |
| **SOFA**^b^ | Cut-off threshold of MIMIC-IV = 6  Cut-off threshold of eICU-CRD = None | - |
| **SAPS-II**^c^ | Cut-off threshold of MIMIC-IV = 6 | - |
| **LR**^d^ | L2 regularization  C = 1 | 100 |
| **KNN**^e^ | K = 5 | - |
| **MLP**^f^ | Optimizer = Adam  Hidden Layer = 100  Learning Rate = 0.001 | 100 |
| **LGBM**^g^ | Extreme profundity of trees = 1  Quantity of helping stages = 450  Learning rate = 0.04 | 100 |
| **RNN**^h^ | Input: 8 h HR, RR, SBP, TEMP  RNN layers = 3  Optimizer = Adam  Learning Rate = 0.001  Cut-off thresholds of RNN  = [2.9, 3, 7.1, 8, 18.2, and 52.8] | 100 |
| **RETAIN^i^** | Input: 12 h HR, RR, SBP, DBP, SpO_2_, TEMP  RNN layers = 1  Optimizer = Adam  Learning Rate = 0.001  Cut-off threshold of RNN = 50 | 100 |

^a^NEWS: national early warning score

^b^SOFA: sequential organ failure assessment

^c^SAPS-II: simplified acute physiology score

^d^LR: logistic regression

^e^KNN: k-nearest neighbors

^f^MLP: multilayer perceptron

^g^LGBM: light gradient boosting method

^h^RNN: recurrent neural network

^i^RETAIN: reverse time attention
