# Supplementary material for: Early Prediction of Cardiac Arrest in the Intensive Care Unit Using Explainable Machine Learning: Retrospective Study
Source: J Med Internet Res. 2024 Sep 17;26:e62890. doi: 10.2196/62890 (PMC11445627; doi:10.2196/62890)
Supplement: Multimedia Appendix 4 [file jmir_v26i1e62890_app4.docx]

**Multimedia Appendix 4.** Demographic information of patients from subgroups of eICU-CRD.

| **Characteristic,**  **Mean (SD^b^)** | **General ICU^a^** | | | **Cardiac ICU** | | |
| --- | --- | --- | --- | --- | --- | --- |
|  | **CA^c^  (n^d^ = 77)** | **Non-CA  (n = 1474)** | ***P* value** | **CA  (n = 106)** | **Non-CA  (n = 3541)** | ***P* value** |
| **Age (year), mean (SD)** | 62.53 (13.91) | 64.47 (15.30) | .39 | 62.53 (13.91) | 64.47 (15.30) | .39 |
| **ICU length of Stay (h),**  **mean (SD)** | 318.90 (346.82) | 273.26 (142.96) | .02 | 216.59 (222.24) | 175.28 (144.14) | .21 |
| **Vital signs,**  **mean (SD)** |  |  |  |  |  |  |
| **HR^e^** | 88.79 (17.60) | 87.10 (17.30) | <.001 | 87.45 (19.54) | 87.35 (17.30) | .41 |
| **RR^f^** | 21.26 (5.68) | 20.99 (5.70) | <.001 | 20.10 (5.90) | 19.76 (4.85) | <.001 |
| **SBP^g^** | 111.21 (22.60) | 118.03 (21.27) | <.001 | 118.88 (21.21) | 125.68 (21.73) | <.001 |
| **DBP^h^** | 59.41 (14.17) | 59.64 (13.70) | <.05 | 63.47 (14.06) | 68.49 (13.86) | <.001 |
| **SpO_2_^i^** | 97.22 (3.57) | 96.92 (2.87) | <.001 | 96.94 (4.01) | 96.55 (2.82) | <.001 |
| **Temperature** | 36.88 (0.91) | 37.12 (0.64) | <.001 | 36.94 (0.94) | 36.90 (0.58) | <.001 |

^a^ICU: intensive care unit

^b^SD: standard deviation

^c^CA: cardiac arrest

^d^n: number of ICU stays

^e^HR: heart rate

^f^RR: respiratory rate

^g^SBP: systolic blood pressure

^h^DBP: diastolic blood pressure

^i^SpO_2_: oxyhemoglobin saturation
