# Supplementary material for: Early Prediction of Cardiac Arrest in the Intensive Care Unit Using Explainable Machine Learning: Retrospective Study
Source: J Med Internet Res. 2024 Sep 17;26:e62890. doi: 10.2196/62890 (PMC11445627; doi:10.2196/62890)
Supplement: Multimedia Appendix 5 [file jmir_v26i1e62890_app5.docx]

**Multimedia Appendix 5.** Statistical comparison of overall the area under the receiver operating characteristic curve between proposed method and baseline methods on the MIMIC-IV.

| **Classifier** | **95% CI**^k^ | | ***P* value** |
| --- | --- | --- | --- |
|  | **Lower limit** | **Upper limit** |  |
| The Proposed Method with FS^a^ vs. NEWS^b^ | .11 | .28 | <.001 |
| The Proposed Method with FS vs. SOFA^c^ | .09 | .26 | <.001 |
| The Proposed Method with FS vs. SAPS-II^d^ | .16 | .33 | <.001 |
| The Proposed Method with FS vs. LR^e^ | .07 | .24 | <.001 |
| The Proposed Method with FS vs. KNN^f^ | .18 | .35 | <.001 |
| The Proposed Method with FS vs. MLP^g^ | .14 | .32 | <.001 |
| The Proposed Method with FS vs. LGBM^h^ | -.01 | .16 | .13 |
| The Proposed Method with FS vs. DEWS^i^ | .18 | .35 | <.001 |
| The Proposed Method with FS vs. RETAIN^j^ | .08 | .26 | <.001 |
| The Proposed Method with FS  vs. The Proposed Method | -.05 | .12 | .90 |

^a^FS: feature screening

^b^NEWS: national early warning score

^c^SOFA: sequential organ failure assessment

^d^SAPS-II: simplified acute physiology score

^e^LR: logistic regression

^f^KNN: k-nearest neighbors

^g^MLP: multilayer perceptron

^h^LGBM: light gradient boosting method

^i^DEWS: deep learning-based early warning score

^j^RETAIN: reverse time attention

^k^CI: confidence interval
