# Supplementary material for: Early Prediction of Cardiac Arrest in the Intensive Care Unit Using Explainable Machine Learning: Retrospective Study
Source: J Med Internet Res. 2024 Sep 17;26:e62890. doi: 10.2196/62890 (PMC11445627; doi:10.2196/62890)
Supplement: Multimedia Appendix 6 [file jmir_v26i1e62890_app6.docx]

**Multimedia Appendix 6.** Statistical comparison of overall event recall between proposed method and baseline methods on the MIMIC-IV.

| **Classifier** | **95% CI**^k^ | | ***P* value** |
| --- | --- | --- | --- |
|  | **Lower limit** | **Upper limit** |  |
| The Proposed Method with FS^a^ vs. NEWS^b^ | -.04 | .30 | .32 |
| The Proposed Method with FS vs. SOFA^c^ | .12 | .45 | <.001 |
| The Proposed Method with FS vs. SAPS-II^d^ | -.08 | .26 | .87 |
| The Proposed Method with FS vs. LR^e^ | -.15 | .18 | .90 |
| The Proposed Method with FS vs. KNN^f^ | .78 | 1.12 | <.001 |
| The Proposed Method with FS vs. MLP^g^ | .48 | .81 | <.001 |
| The Proposed Method with FS vs. LGBM^h^ | .32 | .66 | <.001 |
| The Proposed Method with FS vs. DEWS^i^≥2.9 | -.07 | .26 | .82 |
| The Proposed Method with FS vs. DEWS≥3 | -.07 | .26 | .82 |
| The Proposed Method with FS vs. DEWS≥7.1 | -.02 | .31 | .17 |
| The Proposed Method with FS vs. DEWS≥8 | -.01 | .33 | .09 |
| The Proposed Method with FS vs. DEWS≥18.2 | .00 | .34 | <.05 |
| The Proposed Method with FS vs. DEWS≥52.8 | .14 | .48 | <.001 |
| The Proposed Method with FS vs. RETAIN^j^ | -.14 | .19 | 0.9 |
| The Proposed Method with FS  vs. The Proposed Method | -.17 | .17 | 0.9 |

^a^FS: feature screening

^b^NEWS: national early warning score

^c^SOFA: sequential organ failure assessment

^d^SAPS-II: simplified acute physiology score

^e^LR: logistic regression

^f^KNN: k-nearest neighbors

^g^MLP: multilayer perceptron

^h^LGBM: light gradient boosting method

^i^DEWS: deep learning-based early warning score

^j^RETAIN: reverse time attention

^k^CI: confidence interval
