# Supplementary material for: Early Prediction of Cardiac Arrest in the Intensive Care Unit Using Explainable Machine Learning: Retrospective Study
Source: J Med Internet Res. 2024 Sep 17;26:e62890. doi: 10.2196/62890 (PMC11445627; doi:10.2196/62890)
Supplement: Multimedia Appendix 7 [file jmir_v26i1e62890_app7.docx]

**Multimedia Appendix 7.** Statistical comparison of overall sensitivity between proposed method and baseline methods on the MIMIC-IV.

| **Classifier** | **95% CI**^k^ | | ***P* value** |
| --- | --- | --- | --- |
|  | **Lower limit** | **Upper limit** |  |
| The Proposed Method with FS^a^ vs. NEWS^b^ | .33 | .65 | <.001 |
| The Proposed Method with FS vs. SOFA^c^ | .13 | .46 | <.001 |
| The Proposed Method with FS vs. SAPS-II^d^ | -.18 | .14 | .90 |
| The Proposed Method with FS vs. LR^e^ | .03 | .36 | <.001 |
| The Proposed Method with FS vs. KNN^f^ | .72 | 1.05 | <.001 |
| The Proposed Method with FS vs. MLP^g^ | .70 | 1.02 | <.001 |
| The Proposed Method with FS vs. LGBM^h^ | .55 | .88 | <.001 |
| The Proposed Method with FS vs. DEWS^i^≥2.9 | .29 | .61 | <.001 |
| The Proposed Method with FS vs. DEWS≥3 | .29 | .61 | <.001 |
| The Proposed Method with FS vs. DEWS≥7.1 | .36 | .68 | <.001 |
| The Proposed Method with FS vs. DEWS≥8 | .37 | .70 | <.001 |
| The Proposed Method with FS vs. DEWS≥18.2 | .44 | .76 | <.001 |
| The Proposed Method with FS vs. DEWS≥52.8 | .54 | .87 | <.001 |
| The Proposed Method with FS vs. RETAIN^j^ | -.22 | .11 | .90 |
| The Proposed Method with FS  vs. The Proposed Method | -.18 | .15 | .90 |

^a^FS: feature screening

^b^NEWS: national early warning score

^c^SOFA: sequential organ failure assessment

^d^SAPS-II: simplified acute physiology score

^e^LR: logistic regression

^f^KNN: k-nearest neighbors

^g^MLP: multilayer perceptron

^h^LGBM: light gradient boosting method

^i^DEWS: deep learning-based early warning score

^j^RETAIN: reverse time attention

^k^CI: confidence interval
