# Supplementary material for: Early Prediction of Cardiac Arrest in the Intensive Care Unit Using Explainable Machine Learning: Retrospective Study
Source: J Med Internet Res. 2024 Sep 17;26:e62890. doi: 10.2196/62890 (PMC11445627; doi:10.2196/62890)
Supplement: Multimedia Appendix 8 [file jmir_v26i1e62890_app8.docx]

**Multimedia Appendix 8.** Statistical comparison of overall false alarm rate between proposed method and baseline methods on the MIMIC-IV.

| **Classifier** | **95% CI**^k^ | | ***P* value** |
| --- | --- | --- | --- |
|  | **Lower limit** | **Upper limit** |  |
| The Proposed Method with FS^a^ vs. NEWS^b^ | -.19 | -.05 | <.001 |
| The Proposed Method with FS vs. SOFA^c^ | -.20 | -.06 | <.001 |
| The Proposed Method with FS vs. SAPS-II^d^ | -.20 | -.06 | <.001 |
| The Proposed Method with FS vs. LR^e^ | -.20 | -.06 | <.001 |
| The Proposed Method with FS vs. KNN^f^ | -.25 | -.12 | <.001 |
| The Proposed Method with FS vs. MLP^g^ | -.18 | -.04 | <.001 |
| The Proposed Method with FS vs. LGBM^h^ | -.16 | -.03 | <.001 |
| The Proposed Method with FS vs. DEWS^i^≥2.9 | -.21 | -.08 | <.001 |
| The Proposed Method with FS vs. DEWS≥3 | -.21 | -.08 | <.001 |
| The Proposed Method with FS vs. DEWS≥7.1 | -.21 | -.08 | <.001 |
| The Proposed Method with FS vs. DEWS≥8 | -.22 | -.08 | <.001 |
| The Proposed Method with FS vs. DEWS≥18.2 | -.22 | -.08 | <.001 |
| The Proposed Method with FS vs. DEWS≥52.8 | -.22 | -.08 | <.001 |
| The Proposed Method with FS vs. RETAIN^j^ | -.21 | -.08 | <.001 |
| The Proposed Method with FS  vs. The Proposed Method | -.10 | .04 | .90 |

^a^FS: feature screening

^b^NEWS: national early warning score

^c^SOFA: sequential organ failure assessment

^d^SAPS-II: Simplified acute physiology score

^e^LR: logistic regression

^f^KNN: k-nearest neighbors

^g^MLP: multilayer perceptron

^h^LGBM: light gradient boosting method

^i^DEWS: deep learning-based early warning score

^j^RETAIN: reverse time attention

^k^CI: confidence interval
