# Supplementary material for: Early Prediction of Cardiac Arrest in the Intensive Care Unit Using Explainable Machine Learning: Retrospective Study
Source: J Med Internet Res. 2024 Sep 17;26:e62890. doi: 10.2196/62890 (PMC11445627; doi:10.2196/62890)
Supplement: Multimedia Appendix 9 [file jmir_v26i1e62890_app9.docx]

**Multimedia Appendix 9.** Statistical comparison of overall the area under the receiver operating characteristic curve between proposed method and baseline methods on the eICU-CRD.

| **Classifier** | **95% CI**^j^ | | ***P* value** |
| --- | --- | --- | --- |
|  | **Lower limit** | **Upper limit** |  |
| The Proposed Method vs. NEWS^a^ | .12 | .33 | <.001 |
| The Proposed Method with FS^b^ vs. SAPS-II^c^ | -.03 | .18 | .50 |
| The Proposed Method with FS vs. LR^d^ | -.02 | .19 | .22 |
| The Proposed Method with FS vs. KNN^e^ | .12 | .33 | <.001 |
| The Proposed Method with FS vs. MLP^f^ | -.03 | .18 | .46 |
| The Proposed Method with FS vs. LGBM^g^ | -.09 | .12 | .90 |
| The Proposed Method with FS vs. DEWS^h^ | .14 | .35 | <.001 |
| The Proposed Method with FS vs. RETAIN^i^ | .06 | .27 | <.001 |
| The Proposed Method with FS  vs. The Proposed Method | -.10 | .11 | .90 |

^a^NEWS: national early warning score

^b^FS: feature screening

^c^SAPS-II: Simplified acute physiology score

^d^LR: logistic regression

^e^KNN: k-nearest neighbors

^f^MLP: multilayer perceptron

^g^LGBM: light gradient boosting method

^h^DEWS: deep learning-based early warning score

^i^RETAIN: reverse time attention

^j^CI: confidence interval
