# Supplementary material for: Early Prediction of Cardiac Arrest in the Intensive Care Unit Using Explainable Machine Learning: Retrospective Study
Source: J Med Internet Res. 2024 Sep 17;26:e62890. doi: 10.2196/62890 (PMC11445627; doi:10.2196/62890)
Supplement: Multimedia Appendix 10 [file jmir_v26i1e62890_app10.docx]

**Multimedia Appendix 10.** Statistical comparison of overall event recall between proposed method and baseline methods on the eICU-CRD.

| **Classifier** | **95% CI**^j^ | | ***P* value** |
| --- | --- | --- | --- |
|  | **Lower limit** | **Upper limit** |  |
| The Proposed Method with FS^a^ vs. NEWS^b^ | -.11 | .14 | .90 |
| The Proposed Method with FS vs. SAPS-II^c^ | .18 | .42 | <.001 |
| The Proposed Method with FS vs. LR^d^ | -.11 | .13 | .90 |
| The Proposed Method with FS vs. KNN^e^ | .69 | .93 | <.001 |
| The Proposed Method with FS vs. MLP^f^ | .34 | .58 | <.001 |
| The Proposed Method with FS vs. LGBM^g^ | -.06 | .18 | .90 |
| The Proposed Method with FS vs. DEWS^h^≥2.9 | -.10 | .14 | .90 |
| The Proposed Method with FS vs. DEWS≥3 | -.10 | .14 | .90 |
| The Proposed Method with FS vs. DEWS≥7.1 | -.08 | .16 | .90 |
| The Proposed Method with FS vs. DEWS≥8 | -.08 | .17 | .90 |
| The Proposed Method with FS vs. DEWS≥18.2 | -.04 | .20 | .53 |
| The Proposed Method with FS vs. DEWS≥52.8 | .02 | .26 | <.05 |
| The Proposed Method with FS vs. RETAIN^i^ | -.12 | .12 | .90 |
| The Proposed Method with FS  vs. The Proposed Method | -.12 | .13 | .90 |

^a^FS: feature screening

^b^NEWS: national early warning score

^c^SAPS-II: simplified acute physiology score

^d^LR: logistic regression

^e^KNN: k-nearest neighbors

^f^MLP: multilayer perceptron

^g^LGBM: light gradient boosting method

^h^DEWS: deep learning-based early warning score

^i^RETAIN: reverse time attention

^j^CI: confidence interval
