# Supplementary material for: Early Prediction of Cardiac Arrest in the Intensive Care Unit Using Explainable Machine Learning: Retrospective Study
Source: J Med Internet Res. 2024 Sep 17;26:e62890. doi: 10.2196/62890 (PMC11445627; doi:10.2196/62890)
Supplement: Multimedia Appendix 11 [file jmir_v26i1e62890_app11.docx]

**Multimedia Appendix 11.** Statistical comparison of overall sensitivity between proposed method and baseline methods on the eICU-CRD.

| **Classifier** | **95% CI**^j^ | | ***P* value** |
| --- | --- | --- | --- |
|  | **Lower limit** | **Upper limit** |  |
| The Proposed Method with FS^a^ vs. NEWS^b^ | .14 | .48 | <.001 |
| The Proposed Method with FS vs. SAPS-II^c^ | .12 | .46 | <.001 |
| The Proposed Method with FS vs. LR^d^ | -.03 | .30 | .27 |
| The Proposed Method with FS vs. KNN^e^ | .80 | 1.14 | <.001 |
| The Proposed Method with FS vs. MLP^f^ | .75 | 1.08 | <.001 |
| The Proposed Method with FS vs. LGBM^g^ | .16 | .49 | <.001 |
| The Proposed Method with FS vs. DEWS^h^≥2.9 | .20 | .53 | <.001 |
| The Proposed Method with FS vs. DEWS≥3 | .20 | .53 | <.001 |
| The Proposed Method with FS vs. DEWS≥7.1 | .28 | .61 | <.001 |
| The Proposed Method with FS vs. DEWS≥8 | .29 | .62 | <.001 |
| The Proposed Method with FS vs. DEWS≥18.2 | .38 | .71 | <.001 |
| The Proposed Method with FS vs. DEWS≥52.8 | .53 | .87 | <.001 |
| The Proposed Method with FS vs. RETAIN^i^ | -.17 | .17 | .90 |
| The Proposed Method with FS  vs. The Proposed Method | -.14 | .20 | .90 |

^a^FS: feature screening

^b^NEWS: national early warning score

^c^SAPS-II: simplified acute physiology score

^d^LR: logistic regression

^e^KNN: k-nearest neighbors

^f^MLP: multilayer perceptron

^g^LGBM: light gradient boosting method

^h^DEWS: deep learning-based early warning score

^i^RETAIN: reverse time attention

^j^CI: confidence interval
