# Supplementary material for: Early Prediction of Cardiac Arrest in the Intensive Care Unit Using Explainable Machine Learning: Retrospective Study
Source: J Med Internet Res. 2024 Sep 17;26:e62890. doi: 10.2196/62890 (PMC11445627; doi:10.2196/62890)
Supplement: Multimedia Appendix 13 [file jmir_v26i1e62890_app13.docx]

**Multimedia Appendix 13.** Statistical comparison of overall the area under the receiver operating characteristic curve between proposed method and baseline methods on the MIMIC-IV in general ICU.

| **Classifier** | **95% CI**^i^ | | ***P* value** |
| --- | --- | --- | --- |
|  | **Lower limit** | **Upper limit** |  |
| The Proposed Method vs. NEWS^a^ | -.05 | .34 | .31 |
| The Proposed Method vs. SAPS-II^b^ | .05 | .44 | <.05 |
| The Proposed Method vs. LR^c^ | -.06 | .33 | .46 |
| The Proposed Method vs. KNN^d^ | .02 | .41 | <.05 |
| The Proposed Method vs. MLP^e^ | .04 | .43 | <.001 |
| The Proposed Method vs. LGBM^f^ | -.17 | .22 | .90 |
| The Proposed Method vs. DEWS^g^ | .04 | .43 | <.001 |
| The Proposed Method vs. RETAIN^h^ | -.06 | .33 | .41 |

^a^NEWS: national early warning score

^b^SAPS-II: Simplified acute physiology score

^c^LR: logistic regression

^d^KNN: k-nearest neighbors

^e^MLP: multilayer perceptron

^f^LGBM: light gradient boosting method

^g^DEWS: deep learning-based early warning score

^h^RETAIN: reverse time attention

^i^CI: confidence interval
