# Supplementary material for: Early Prediction of Cardiac Arrest in the Intensive Care Unit Using Explainable Machine Learning: Retrospective Study
Source: J Med Internet Res. 2024 Sep 17;26:e62890. doi: 10.2196/62890 (PMC11445627; doi:10.2196/62890)
Supplement: Multimedia Appendix 15 [file jmir_v26i1e62890_app15.docx]

**Multimedia Appendix 15.** Statistical comparison between Gini index-based feature of SpO_2_ and top 25 global importance features in the proposed method.

| **Feature name** | **t-value** | ***P value* (Uncorrected)** | ***P* value**  **(FDR^l^ corrected)** | **Global importance feature** |
| --- | --- | --- | --- | --- |
| EWS^a^_SpO_2_^b^_1_6h_Min^c^ | 22.28 | <.001 | <.001 | O |
| TEMP^d^_2h | -8.54 | <.001 | <.001 | O |
| HR^e^_5_8_Skewness | 5.87 | <.001 | <.001 | O |
| EWS_RR^f^_12h | -2.26 | .024 | .41 | O |
| SpO_2__1_4h_Skewness | 1.01 | .31 | .50 | O |
| EWS_RR_9h | 22.57 | <.001 | <.001 | O |
| TEMP_1_6h_Max^g^ | -5.52 | <.001 | <.001 | O |
| Temp_1_12h_Max | 8.09 | <.001 | <.001 | O |
| SpO_2__5_8h_Min | 18.22 | <.001 | <.001 | O |
| EWS_RR_1_4h_Max | -4.69 | <.001 | <.001 | O |
| HR_7_12h_IQR^h^ | -7.44 | <.001 | <.001 | O |
| EWS_9_12h_Max | -5.13 | <.001 | <.001 | O |
| EWS_1_4h_Median | 1.05 | .30 | .62 | O |
| HR_11h | 1.02 | .31 | .62 | O |
| DBP^i^_9_12h_Skewness | -3.16 | <.05 | <.05 | O |
| EWS_HR_8h | -3.76 | <.001 | <.05 | O |
| EWS_SBP^j^_9_12h_Max | 3.51 | <.001 | <.05 | O |
| EWS_SpO2_2h | -5.10 | <.001 | <.001 | O |
| EWS_5_8h_Min | -2.00 | <.05 | .62 | O |
| RR_5_8h_Median | -5.17 | <.001 | <.001 | O |
| RR_1_12h_Skewness | -5.69 | <.001 | <.001 | O |
| EWS_TEMP_9_12h_Min | 1.99 | <0.05 | .63 | O |
| EWS_SpO_2__5_8h_Min | -5.67 | <.001 | <.001 | O |
| TEMP_1_6h_Skewness | -6.80 | <.001 | <.001 | O |
| SBP_1_6h_Std^k^ | 17.14 | <.001 | <.001 | O |
| Gini_SpO_2_ | -35.75 | <.001 | <.001 | X |

^a^EWS: early warning score

^b^SpO_2_: oxyhemoglobin saturation

^c^Min: minimum

^d^TEMP: temperature

^e^HR: heart rate

^f^RR: respiratory rate

^g^Max: maximum

^h^IQR: interquartile range

^i^DBP: diastolic blood pressure

^j^SBP: systolic blood pressure

^k^Std: standard deviation

^l^FDR: false discovery rate
